# Endolymphatic duct blockage for Ménière’s disease: a double-blind, randomised controlled trial

**DOI:** 10.1016/j.lanepe.2026.101765

**Published:** 2026-07-06

**Authors:** Annejet A. Schenck, Raymond van de Berg, Thomas T.A. Peters, Yvette E. Smulders, Stephanie M. Winters, Stefan Böhringer, Adriaan F. Holm, Jérôme J. Waterval, Sebastiaan Hammer, Susan P.M. Hombergen, Milou R. Munting, Peter Paul G. van Benthem, Josephina M. Kruyt, Hendrikus M. Blom

**Affiliations:** a Department of Otorhinolaryngology and Head & Neck Surgery, Leiden University Medical Centre, Leiden, the Netherlands; b Department of Otorhinolaryngology, Haga Hospital, The Hague, the Netherlands; c Department of Otorhinolaryngology and Head and Neck Surgery, School for Mental Health and Neuroscience (MHeNS), Maastricht University Medical Centre, Maastricht, the Netherlands; d Department of Otorhinolaryngology, Frisius Medical Centre, Leeuwarden, the Netherlands; e Department of Otorhinolaryngology, Beatrixziekenhuis Rivas Zorggroep, Gorinchem, the Netherlands; f Department of Otorhinolaryngology, Gelre Hospitals, Apeldoorn, the Netherlands; g Department of Medical Statistics, Leiden University Medical Centre, Leiden, the Netherlands; h Department of Clinical Pharmacology and Toxicology, Leiden University Medical Centre, Leiden, the Netherlands; i Department of Otorhinolaryngology, Wilhelmina Hospital Assen, Assen, the Netherlands; j Department of Radiology, Haga Hospital, The Hague, the Netherlands; k Department of Physiotherapy, Haga Hospital, The Hague, the Netherlands; l Department of Otorhinolaryngology, Antwerp University Hospital, Antwerp, Belgium

**Keywords:** Ménière’s disease, Endolymphatic duct blockage, Endolymphatics sac decompression, Surgery, Vertigo, Randomized controlled trial

## Abstract

**Background:**

Treatment for Ménière’s disease remains a subject of debate. A surgical technique in which the endolymphatic duct is blocked was proposed as a new treatment modality for patients with intractable Ménière’s disease, but a double-blind trial was lacking. Therefore, the aim of this double-blind trial was to assess whether EDB is more effective than ESD in patients with intractable Ménière’s disease.

**Methods:**

This is a double-blind, randomised, multicentre trial comparing endolymphatic duct blockage (EDB) to endolymphatic sac decompression (ESD). Patients had unilateral, active Ménière’s disease despite treatment with at least two corticosteroid injections. All patients were recruited from two university and five non-university hospitals in the Netherlands. During surgery, patients were randomly assigned to either the EDB or ESD group. Following surgery, patients were followed for 1 year, with both physical visits and an app in which attacks could be reported. Both patients and investigators were blinded throughout the follow-up period. The primary outcome was freedom from vertigo attacks at 12 months, defined as no attacks during the preceding 6 months. Secondary outcome measures included attack incidence, quality of life, dizziness, tinnitus, and inner ear function. Analyses were performed according to the intention-to-treat principle. This trial was registered in the ISRCTN registry (registered 24-02-2021, https://www.isrctn.com/ISRCTN12074571).

**Findings:**

Between 23 June 2021 and 12 September 2023, 75 patients with definite Ménière’s disease were enrolled; 39 underwent EDB, while 36 underwent ESD. No loss to follow-up was recorded. At 12 months, 23/39 (59%) patients in the EDB group and 24/36 (67%) in the ESD group were free from vertigo attacks (OR 0.72, 95% CI 0.28–1.84; p = 0.65). Secondary outcomes, including quality of life, dizziness, tinnitus, and hearing function did not differ between groups. Higher baseline patients expectations were associated with treatment success. Three serious adverse events occurred (one in the EDB group, two in the ESD group) and no deaths were reported.

**Interpretation:**

This trial does not demonstrate a benefit of EDB over ESD for patients with refractory Ménière’s disease. Given the comparable outcomes between groups, EDB should not be preferred over ESD. The association between baseline expectations and outcome suggests that non-specific treatment effects may contribute to perceived benefit. Future studies should consider inclusion of a non-surgical or sham-surgery control to more definitely determine the effect of this type of surgery, although such trials would be challenging.

**Funding:**

Dutch National Healthcare Institute and ZonMw (‘Veelbelovende Zorg’ grant).

**Research in context**

**Evidence before this study**

We searched PubMed, Embase, and the Cochrane Library for studies published up to June, 2026, using terms related to “Ménière’s disease”, “endolymphatic duct blockage”, and “endolymphatic sac surgery”. Endolymphatic sac surgery is widely used, with reported vertigo control rates of around 60–70%, but its effectiveness remains debated, particularly after sham-controlled data suggested a substantial placebo effect. Endolymphatic duct blockage (EDB) has been proposed as a novel technique, with one non-blinded randomised trial reporting very high efficacy (>95%). However, existing studies are limited by lack of blinding, small sample sizes, and potential bias. High-quality double-blind evidence has been lacking.

**Added value of this study**

In this multicentre, double-blind, randomised controlled trial, we found no significant difference between EDB and endolymphatic sac decompression (ESD) in freedom from vertigo attacks at 12 months or in secondary outcomes. Approximately two-thirds of patients in both groups were free from attacks. Patient expectations were associated with treatment success. These findings challenge previous reports of large treatment effects of EDB.

**Implications of all the available evidence**

This trial does not demonstrate a clinically meaningful benefit of EDB over ESD. Improvements after surgery may at least partly reflect non-specific effects and the natural course of Ménière’s disease. Careful counselling and shared decision making are essential. Future studies should consider sham-controlled or non-surgical comparisons to better determine true treatment effects.

## Introduction

Ménière’s disease (MD) is a disorder of the inner ear, characterised by vertigo attacks with tinnitus, aural fullness, and fluctuating sensorineural hearing loss. It was first described in 1861 by Prosper Menière,^1^ who initially considered it a neurological disease. Current evidence suggests that hydrops of the endolymph in the inner ear is related to the symptoms of MD. However, the aetiology and pathophysiology of the disease remain unclear, making it difficult to decide on management strategies.

Various treatment modalities have been suggested, such as diets, drugs, intratympanic injections, and surgery; however, the effectiveness of these techniques is very uncertain.^2^, ^3^, ^4^, ^5^ Another option is surgical intervention, which can be either destructive, such as selective vestibular neurectomy and labyrinthectomy, or non-destructive. Destructive surgeries effectively terminate vertigo attacks^6^ but at the cost of complete loss of function of the inner ear. Moreover, MD may occur bilaterally. Therefore, these destructive procedures should only be considered in cases of intractable disease or in patients with non-serviceable hearing. The first non-destructive surgical intervention was described in 1927 by Portmann.^7^ He hypothesised that pressure in the endolymphatic sac, caused by a surplus of endolymph, leads to attacks. Therefore, he designed a procedure to relieve the pressure while retaining inner ear function. In this procedure, the mastoid bone over the endolymphatic sac was removed; hence, it was named ‘endolymphatic sac decompression’ (ESD). Later, similar procedures involving the endolymphatic sac were proposed, such as sac shunting and drainage after decompression, collectively called ‘endolymphatic sac surgery’ (ESS). Notably, most interventions lead to a reduction of complaints in approximately 70% of the patients; however, the contribution of the placebo effect and/or natural course of the disease to this success rate is still a subject of debate.^8^, ^9^, ^10^, ^11^

In 2015, a non-blinded trial was published, assessing another new technique that focuses on the endolymphatic space. In this procedure, the endolymphatic duct (which connects the endolymphatic sac to the rest of the inner ear) is blocked using a clip. The trial compared patients who had undergone this endolymphatic duct blockage (EDB) with a control group of patients who had undergone ESD.^12^ The results of EDB operation in this trial were promising, with over 95% of the patients still free from attacks 2 years after surgery vs. only 30% of those in the ESD group. However, this trial was not blinded, and the effect of ESD was low compared to that in previous studies, in which ESD was effective in approximately 70% of the patients.^13^^,^^14^ Given these circumstances, a double-blind repetition of the trial was required to generate conclusive evidence for the effectiveness of the EDB technique.

Therefore, we conducted this double-blind, prospective, randomised controlled trial to evaluate the effect of EDB by comparing it to that of ESD.

## Methods

### Study design and ethical approval

This multicentre, randomised, controlled, double-blind, parallel-group trial was performed in two university and five non-university hospitals in the Netherlands. The study protocol was approved by the local medical ethics committee of Leiden-Den Haag-Delft (METC LDD, number P20.118, date of approval 19-03-2021), a board of experts of the Dutch National Health Care Institute (decision number: 2020010440), the local research committee of the HagaHospital (number T20.108), and the patient association for inner ear disease (Stichting Hoormij). Moreover, trial participation was approved by the boards of all participating centres. In accordance with national regulations, the study was monitored by a study monitor. A data safety monitoring board was appointed, and they met three times to discuss safety aspects. The full protocol of this trial was published before patients were enrolled in the study.^15^

### Participants

We recruited patients with unilateral, definite MD according to the Bárány Society criteria,^16^ who experienced vertigo attacks despite treatment with intratympanic steroids. The inclusion criteria are presented in Supplementary Table S1.

Patients were informed about the trial both verbally and in written form. Within 2 weeks, patients received further explanation and were given the opportunity to ask questions. All patients provided written and verbal informed consent for participation. After signing, patients underwent extensive testing and filled in several questionnaires. Surgery was performed preferably within 8 weeks.

### Randomisation and masking

Patients were randomly, electronically assigned to either the EDB or ESD (control treatment) group by an automated randomisation service provided by Castor (Castor v2025.3.0.0, Amsterdam, The Netherlands). Randomisation was stratified based on site of inclusion, sex (male/female), and duration of disease (new onset/mature). MD was categorised as ‘recent onset’ if the first vertigo attack occurred less than 2 years before inclusion and ‘mature’ if patients had been suffering from the disease for over 2 years. Block sizes of two or four per inclusion site were used and allocation ratio was 1:1. Randomisation was performed in the operating theatre (OT).

The allocation was concealed, and no electronic implant registration was performed; this information was kept on paper throughout the trial. No visible difference was observed on the wound or scar after surgery between the groups. No one present in the OT during surgery saw the patient after the operation or later during follow-up. The investigator in charge of the follow-up was not present during surgery and could not in any way retrieve allocation. Logging was used in the electronic data capturing system, which also registered patients’ randomisation. Patients were not deblinded until the last patient had completed follow-up in November 2024.

### Researchers

Patients were recruited in seven hospitals. Every hospital had a local investigator, who was trained by the research team on informing and including patients. These local investigators were not present during surgery and conducted the follow-up. All the information obtained was sent electronically to the coordinating researcher (AS), who entered the data into the study database. The researcher was blinded to the allocation throughout the trial. The statistician (SB) was blinded when developing the analysis scripts.

### Procedures

All patients underwent surgery. After a regular canal wall up mastoidectomy, the endolymphatic sac was identified and skeletonised. All bones over the endolymphatic sac were removed to enable insertion of the clip instrument. Then, an open clip was placed over the endolymphatic duct, and a computed tomography scan was acquired to assess the clip’s position. Randomisation was performed when radiological confirmation showed that the clip was positioned correctly (at the ostium of the vestibular aqueduct). For patients in the EDB group (the intervention group), the clip was closed such that the duct was blocked, whereas in the ESD group, the (open) clip was removed. The mastoid was not obliterated to maintain decompression of the sac, and the skin was closed in layers. The method of EDB surgery was described in detail (including videos) in another publication.^17^ One surgeon with extensive experience in this technique (HB) was present during all surgeries to rule out surgical variation.

After surgery, patients were followed up for 1 year. All patients used an app in which they completed a questionnaire every evening and could immediately report attacks. They visited the clinic four times: 1 week after surgery for wound inspection and 3, 6, and 12 months following the operation. Throughout this period, both patient and clinician were blinded. Moreover, patients received three sessions of individually tailored vestibular rehabilitation therapy from experienced vestibular therapists every 2 weeks, starting 1 week after surgery. All tests performed at each moment of follow-up are listed in Supplementary Table S2.

### Outcomes

The primary outcome measure was freedom from vertigo attacks after 12 months of follow-up, measured as proportion of patients per group. Patients were considered free from attacks if they had not experienced any attacks for 6 months before the 12-month follow-up visit. The occurrence of attacks was established during the follow-up visits and then dichotomised (yes/no). The exact number of attacks was not discussed during the visits to avoid recall bias.

Secondary outcome measures were as follows:

- Number of days with reported attack(s), counted by using data from the DizzyQuest app.^18^
- Severity of attacks, measured using the DizzyQuest app, on a scale of 1 (very mild) to 7 (extremely severe). Per patient, an average severity score was calculated.
- Quality of life, measured using the Functional Level Scale (FLS), the 36-Item Short Form Health Survey (SF-36, Dutch norm-based standardisation, physical and mental domain), EuroQol 5-Dimension questionnaire (EQ-5D, five level, utility index scores using the Dutch EQ-5D-5L tariff), visual analogue scale (VAS, score 0–100) questionnaires.
- Hearing, measured using pure tone audiometry (PTA), with the mean hearing loss in decibels at 0.5, 1, 2 and 3 kHz, in which 3 kHz was computed from averaging 2 and 4 kHz. Moreover, speech discrimination score was used, defined as correctly repeated monosyllabic words from a standardized word list presented under controlled listening conditions.
- Vestibular function, measured with caloric testing by means of slow phase velocity. This was calculated per side by summing the slow phase velocity of both warm and cold water.
- Dizziness, assessed using the Dizziness Handicap Inventory (DHI).
- Tinnitus, assessed using the Tinnitus Handicap Inventory (THI).
- Use of escape medication and/or co-intervention.
- Complications of surgery.

Patients also filled out a questionnaire about expectations from the surgery on a 4-item scale to assess correlation with outcomes (scale 0–3), in which a higher score indicates more positive expectations. The average score was used as indicator for patient expectation. The association between baseline patient expectations and the primary outcome was assessed using logistic regression analysis. In addition, Spearman’s rank correlation was used to evaluate the association between expectations and app-reported attack frequency.

All data obtained were centrally assessed. Adverse events were registered in the patient’s electronic file. Serious adverse events (SAEs) were reported to the medical ethics committee without any delay. The committee assessed each SAE and discussed it to determine the consequences for the trial, such as stopping.

### Statistical analysis

This study was designed as a superiority trial to detect a difference in the proportion of patients free from vertigo attacks between groups. The sample size calculation was based on the only prospective study on EDB,^12^ in which 96 .5% of the patients who underwent EDB achieved complete control of vertigo. The anticipated effect of ESD was 70% success, derived from earlier research.^13^^,^^19^ Sample size was therefore calculated for vertigo control in 95% of patients in the EDB group compared to 70% in the control group (power of 80%) for 1:1 allocation. Therefore, the null hypothesis was that the difference in the primary outcome measure is zero, with a two-sided alternative hypothesis of a difference between the groups. The risk of a false positive finding is controlled at the 5% level. To obtain a power of at least 80% for Fisher’s Exact test, the required sample size was 32 per group. We also anticipated a loss to follow-up of 10 patients per group, leading to 42 patients per group and a total group size of 84 patients to achieve the required power. These numbers assume a uniform attrition rate of 23.8%.

All analyses were performed following an intention-to-treat approach. Binary outcomes were analysed using the Chi–Square test, and continuous outcomes were compared using a t-test. Longitudinal data were analysed using the *lmer* function of the *lme4* package in R. Models contain a random intercept and slope per patient and use baseline measurements as covariate. Missing follow-up measurements were ignored, thereby using a missing-at-random assumption. Both a main effect for randomisation group as well as a time × group interaction was included in the models. Time was treated as numeric value (assuming a linear trend). Linearity of time was checked graphically using mean profile plots.

Analyses for changes in quality of life (SF36, EQ-5D, VAS) and DHI score were carried out by first categorising into decreased, no change, or increased score at 12 months vs. baseline for both groups, which gave rise to a 3 × 2 table. These tables were tested using a Chi–Square test. Logistic regression analysis was performed for the primary outcome, the app-based outcome and correlation of primary outcome and patient expectations. Effect size is reported as OR.

Complete case analysis was performed when missing data were present. All analyses were performed using R software, version 4.3.3 using reproducible R-markdown files.

### Role of the funding source

The funder of the study (Dutch National Healthcare Institute and ZonMw) had no role in data collection, analysis, and interpretation, or writing of the manuscript. An annual meeting with the funder was organised to discuss progress of the trial; no outcomes were discussed during this meeting.

## Results

### Participants

**Fig. 1:**
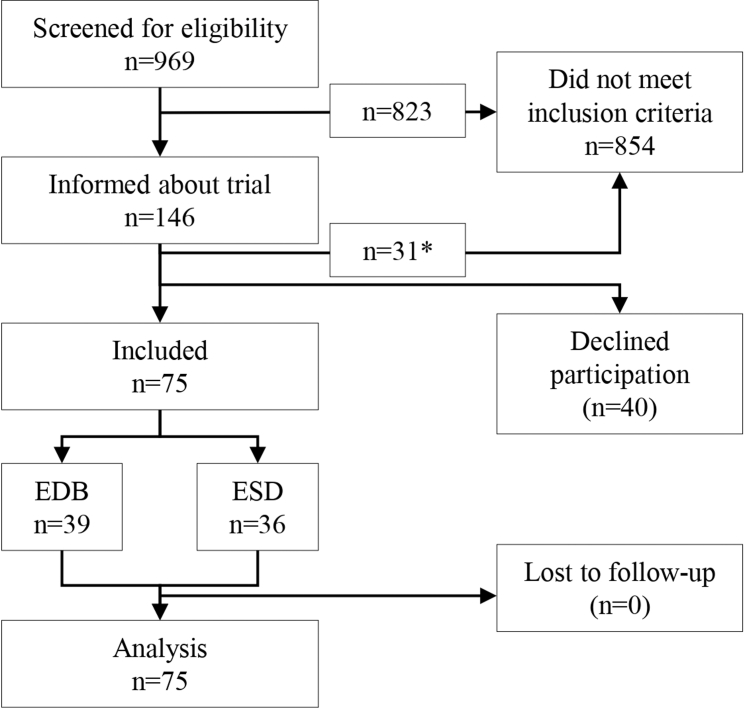
**Patients’ flowchart.** Abbreviations: EDB = endolymphatic duct blockage, ESD = endolymphatic sac decompression. ∗Some patients were informed about the trial, but did not suffer attacks in the weeks after counselling. Because patients had to meet the criteria of ‘one attack in the last two months’, some patients did not anymore meet the criteria at the moment of decision, even if they had initially been eligible.

The first patient was enrolled on 23 June 2021. Eighteen months after enrolment began, none of the patients were lost to follow-up, whereas the anticipated dropout was 20 participants. The target sample size was therefore reduced from 84 to 74 patients (anticipating 10 dropouts instead of 20). This decision was made without inspection of treatment outcome and was approved as amendment to the protocol by the medical ethics committee (decision number P20.118/MS/ms, 12-04-2023). In total, 75 patients were included in the trial, as the final two patients were enrolled on the same day (September 12, 2023). No participants were lost to follow-up during the rest of the trial and therefore, sample size remained adequate. The patients’ flowchart is shown in Fig. 1, and baseline characteristics are presented in Table 1. Most patients had experienced MD for more than 2 years, with a slight preponderance of male participants.

**Table 1:** Baseline characteristics of the intention-to-treat population.

	EDB (n = 39)	ESD (n = 36)
Sex		
Male	20/39 (51%)	18/36 (50%)
Female	19/39 (49%)	18/36 (50%)
Side of disease		
Left	16/39 (41%)	15 (42%)
Right	23/39 (59%)	21 (58%)
Age at inclusion (years)	56 (13)	58 (11)
Duration of disease (years)	7.0 (6.3)	5.7 (4.9)
Recent (<2 years)	6/39 (15%)	6/36 (17%)
Mature (>2 years)	33/39 (85%)	30/36 (83%)
Number of attacks		
Last 2 months	6.8 (6.5)	7.1 (7.4)
Last 6 months	18.2 (15.8)	17.2 (12.2)
Quality of life		
FLS	4.2 (0.9)	3.9 (1.1)
SF-36, physical component summary	40.7 (7.8)	43.8 (7.4)
SF-36, mental component summary	40.6 (6.9)	41.0 (6.6)
EQ-5D	0.86 (0.11)	0.87 (0.11)
VAS	61.2 (17.1)	66.4 (18.5)
DHI	61.7 (18.4)	50.7 (18.2)
THI	45.0 (24.9)	32.8 (22.4)
Hearing		
PTA affected ear (dB)	54.5 (17.4)	50.6 (14.0)
SDS affected ear (%)	74.6 (32.6)	78.2 (17.8)
PTA unaffected ear (dB)	18.6 (9.6)	17.2 (13.7)
SDS unaffected ear (%)	99.0 (4.4)	98.2 (7.8)
Vestibular function (mean slow phase velocity, °/sec)		
Affected ear	10.7 (8.5)	9.2 (6.9)
Unaffected ear	14.4 (11.5)	15.0 (13.3)

Values are mean (SD).

EDB = endolymphatic duct blockage, ESD = endolymphatic sac decompression, FLS = functional level scale, SF36 = short form 36, EQ5D = EuroQol 5 domains questionnaire, PTA = pure tone audiometry, SDS = speech discrimination score, dB = decibel.

### Primary outcome

At 12 months, 23/39 (59%) patients in the EDB group and 24/36 (67%) in the ESD group were free from attacks (risk difference −7.7 percentage points, 95% CI −29.7 to 14.3; OR 0.72, 95% CI 0.28 to 1.84; p = 0.65).

### Vertigo attack frequency

For counting of vertigo attacks, all patients had the DizzyQuest app on their phone from (at latest) the day of surgery until at least 365 days after surgery. The button to report an attack was hit 1094 times. After exclusion of empty entries, reports outside the follow-up period (before the date of surgery or >12 months after surgery), and duplicate entries on the same day, 660 days with one or more attacks remained, reported by 56 patients.

Based on these app-derived data, 26/39 (66.7%) patients in the EDB group and 15/36 (41.7%) patients in the ESD group were classified as attack-free, which differed significantly between the groups (risk difference 25.0 percentage points, 95% CI 2.7 to 47.3; odds ratio 2.8, 95% CI 1.1 to 7.2, p = 0.03). Mean attack severity did not differ between groups (5.1 in the EDB group vs. 4.7 in the ESD group, absolute difference −0.4, 95% CI −1.0 to 0.2, p = 0.15).

In the EDB group (39 patients; 14,235 follow-up days), attacks were reported on 288 days (mean proportion 2.0% of days, SD 3.0, range 0–55). In the ESD group (36 patients; 13,140 follow-up days), attacks were reported on 372 days (2.8% of days; SD 3.1, range 0–41). The between-group difference was −0.8 percentage points (95% CI −0.6 to 2.2; p = 0.26). The median number of days with an attack per patient was 3.0 days (IQR 0.0–12.0) in the EDB group vs. 3.5 days (IQR 1.3–17.0) in the ESD group (Mann–Whitney U = 563, p = 0.14). There was no correlation between patient expectations and the number of attacks reported via the app (Spearman’s ρ = −0.12; 95% CI −0.35 to 0.11, p = 0.33).

### Quality-of-life outcomes

**Fig. 2:**
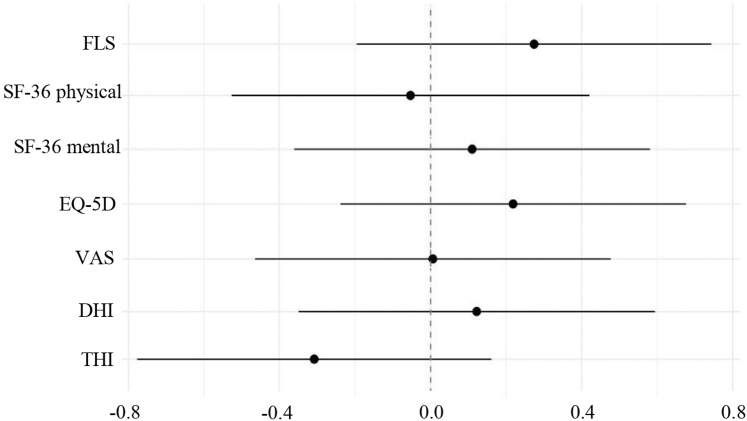
**Forest plot of standardized between-group effects with 95% confidence intervals.** Standardized mean differences are shown for FLS, SF-36 physical, SF-36 mental, EQ-5D, VAS, DHI, and THI. Points indicate the effect estimates, and horizontal lines represent 95% confidence intervals. The vertical dashed line denotes no effect (standardized mean difference = 0). Standardized differences were calculated by averaging standard deviations across both groups and dividing differences and CI limits by this standard deviation. Abbreviations: FLS = functional level scale, SF-36 = the 36-Item Short Form Health Survey, EQ-5D = EuroQol 5-Dimension questionnaire, VAS = visual analogue scale, DHI = Dizziness Handicap Inventory, THI = Tinnnitus Handicap Inventory.

No between-group differences were observed for any quality-of-life outcome at 12 months (Table 2). Changes over time in the Dizziness Handicap Inventory (DHI) and Tinnitus Handicap Inventory (THI) did not differ between the groups (Fig. 2; Table 2).

**Table 2:** Between-group differences at 12 months.

	EDB	ESD	Between−group difference	95% CI	p−value
Free from attacks (primary outcome)	23/39 (59%)	24/36 (67%)	−7.7 percentage points	−29.7 to 14.3	0.65
App−based attack−free	26/39 (67%)	15/36 (42%)	25.0 percentage points	2.7 to 47.3	0.03
Percentage of days with attack(s)	2.0 (3.0)	2.8 (3.1)	0.8 percentage points	−0.6 to 2.2	0.26
Number of days with attack(s)^a^	3.0 (0.0−12.0)	3.5 (1.3−17.0)	–	–	0.14
Attack severity	5.1 (1.1)	4.7 (1.0)	−0.4	−1.0 to 0.2	0.15
FLS	2.7 (1.6)	3.1 (1.1)	0.4	−0.3 to 1.0	0.25
SF−36 physical	46.0 (8.7)	45.6 (8.0)	−0.4	−4.4 to 3.5	0.82
SF−36 mental	43.1 (6.8)	43.8 (5.5)	0.7	−2.2 to 3.6	0.64
EQ−5D	0.87 (0.15)	0.90 (0.09)	0.03	−0.03 to 0.08	0.34
VAS	69.9 (25.5)	70.1 (20.5)	0.1	−10.8 to 11.0	0.98
DHI	33.2 (26.5)	36.2 (22.8)	3.0	−8.7 to 14.7	0.61
THI	34.0 (27.7)	26.6 (19.2)	−7.3	−18.5 to 3.8	0.19
Hearing (PTA, affected ear)	48.0 (22.5)	51.4 (18.3)	3.4	−6.3 to 13.0	0.49
Hearing (SDS, affected ear)	80.9 (27.6)	65.7 (32.3)	−15.2	−30.5 to 0.1	0.051
Vestibular function (affected ear)	8.2 (6.4)	9.7 (7.0)	1.5	−1.9 to 5.0	0.38

Data are presented as n/N (%), mean (SD), or median (IQR), as appropriate.

EDB = endolymphatic duct blockage. ESD = endolymphatic sac decompression. CI = confidence interval. Attack severity: range 1–7, higher score indicates more severe attack. FLS=Functional Level Scale (range 1–6, higher scores indicate greater functional impairment). SF-36 = 36-Item Short Form Health Survey physical and mental component summary scores (norm-based scores; higher scores indicate better health status). EQ-5D = EuroQol 5-Dimension questionnaire utility score (range 0–1, higher scores indicate better health-related quality of life). VAS = visual analogue scale for self-rated health (range 0–100, higher scores indicate better health status). DHI = Dizziness Handicap Inventory (range 0–100, higher scores indicate greater dizziness-related disability). THI=Tinnitus Handicap Inventory (range 0–100, higher scores indicate greater tinnitus-related handicap). PTA = pure-tone average hearing threshold. SDS = speech discrimination score (% correctly repeated monosyllabic words). Vestibular function: sum of caloric-induced slow-phase velocities (warm and cold irrigation) on the affected side.

a Median (IQR) are reported because of skewed distributions.

### Hearing and vestibular outcomes

**Fig. 3:**
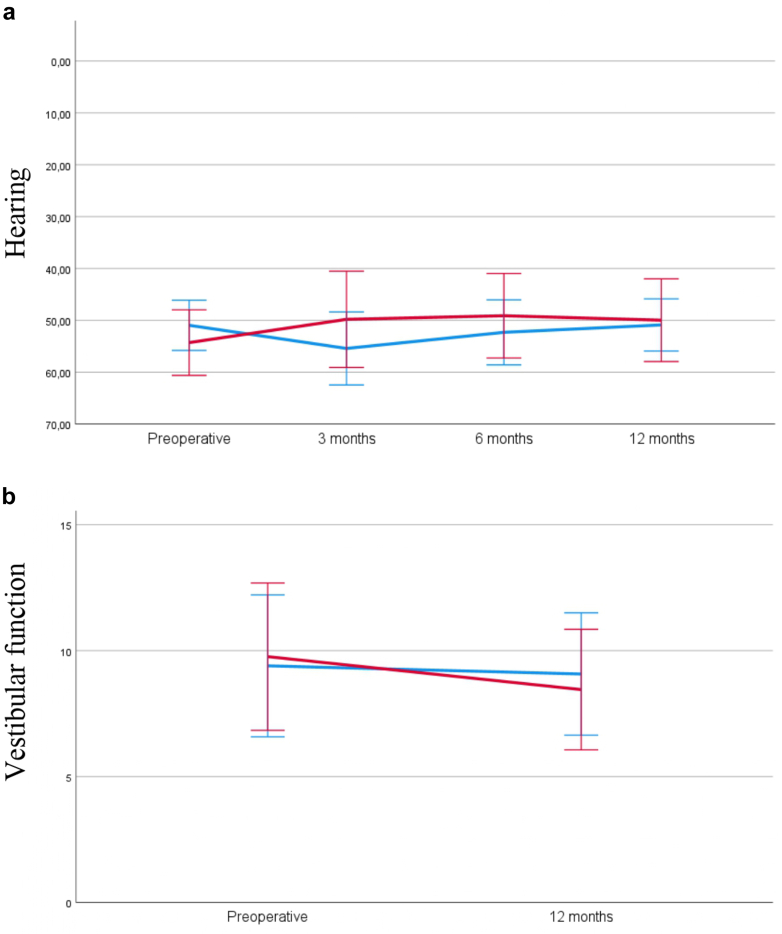
**Hearing and vestibular function throughout the trial in the affected (operated) ear.** (a) Hearing thresholds over time. The y-axis shows mean hearing loss in decibels, calculated as the pure-tone average at 0.5, 1, 2, and 3 kHz, where 3 kHz was estimated by averaging thresholds at 2 and 4 kHz. (b) Vestibular function over time. The y-axis shows vestibular function assessed by caloric testing, expressed as the summed slow-phase velocity for warm and cold water irrigations. Mean values are shown with standard deviation error bars. No significant between-group differences were observed at 12 months for hearing (mean difference PTA 3.4, 95% CI −6.3 to 13.0, p = 0.49) or vestibular function (difference 1.5, 95% CI −1.9 to 5.0, p = 0.38). Abbreviations: PTA = pure tone audiometry.

Mean hearing thresholds of the affected ear remained stable after surgery (EDB: 54.5 to 48.0 dB; ESD: 50.6 to 51.4 dB) (Fig. 3). No significant difference was observed between the groups in PTA after 12 months of follow-up (between-group difference 3.4 dB, 95% CI −6.3 to 13.0, p = 0.49) or speech discrimination (between group difference −15.2, 95% CI −30.5 to 0.1, p = 0.051). Postoperative vestibular function did not differ between groups (between-group difference 1.5, 95% CI −1.9 to 5.0, p = 0.38) (Fig. 3).

Descriptive statistics for all patient-reported, audiometric, and vestibular outcome measures at each study timepoint are shown in Supplementary Table S3.

### Additional treatments

Additional treatment was categorised as medication, intratympanic injections with corticosteroids, intratympanic injections with gentamicin, or destructive surgery (Table 3). No statistical difference was observed between the groups in any category of additional treatment. None of the patients underwent destructive surgery during the course of the trial. Five patients underwent intratympanic injection with gentamicin because of unbearable complaints. One of these patients had undergone ESD; the other four had undergone EDB.

**Table 3:** Overview of additional treatment per group per follow-up interval.

	EDB-group (n = 39)	ESD-group (n = 36)	p-value
Surgery-1 week			
Medication^a^	3	0	0.24
ITC	0	0	–
ITG	0	0	–
Destructive surgery	0	0	–
1 week–3 months			
Medication^a^	3	0	0.24
ITC	2	5	0.25
ITG	1	0	–
Destructive surgery	0	0	–
3–6 months			
Medication^a^	2	0	0.49
ITC	2	6	0.14
ITG	0	1	0.48
Destructive surgery	0	0	–
6–12 months			
Medication^a^	3	1	0.62
ITC	6	5	1.0
ITG	3	0	0.24
Destructive surgery	0	0	–

EDB = endolymphatic duct blockage, ESD = endolymphatic sac decompression, ITC = intratympanic injections with corticosteroids, ITG = intratympanic injection with gentamicin.

a Only vertigo-related medication was registered. It concerned either betahistine of cinnarizine.

### Safety outcomes

Serious adverse events occurred in 1/39 (3%) EDB patients and 2/36 (6%) ESD patients. One patient suffered persistent cerebrospinal fluid leakage, one suspected intracranial infection, and one transient complete vestibulocochlear loss. The first patient recovered after revision surgery, the second patient recovered completely after treatment with antibiotics, and the third patient experienced spontaneous recovery. Benign paroxysmal positional vertigo occurred in 10 patients: seven in the EDB group and three in the ESD group. All patients were treated with the Epley manoeuvre, which relieved complaints.

### Expectation analysis

Patients filled in a questionnaire about their expectations for the surgery. A score of 0 or 1 meant ‘strongly disagree’ and ‘disagree’, respectively. A score of 2 or 3 meant ‘agree’ or ‘strongly agree’. Notably, most patients (57/75, 76%) strongly believed (score 3) that surgery would relieve their complaints. In addition, 16/75 (21%) scored 2 points. The mean score of all patients was 2.6 (SD 0.6) on a scale of 0–3. This expectation correlated significantly with the primary outcome (odds ratio 3.3, 95% CI 1.2 to 9.1, p = 0.021). This was the only baseline characteristic that was predictive of the eventual outcome. Correction for age, gender, and duration of disease did not alter this result.

## Discussion

In this double-blind, randomised trial, no significant difference was observed between endolymphatic duct blockage (EDB) and endolymphatic sac decompression (ESD) in the proportion of patients free from vertigo attacks at 12 months after surgery. Secondary outcomes, including quality of life, inner ear function, and the need for additional treatment were also similar between the groups. Patient expectation prior to surgery was the only variable associated with the primary outcome.

Endolymphatic duct blockage (EDB) is a relatively new surgical procedure, first performed on humans in 2015, and remains an area of ongoing clinical debate due to limited high-quality evidence.^12^ Most published data consist of small retrospective series reporting heterogeneous outcomes, limiting comparability between studies.^20^, ^21^, ^22^, ^23^, ^24^, ^25^ The only prospective randomised study reported high success rates for EDB compared with ESD,^12^; however, its non-blinded design made it susceptible to placebo and nocebo effects. Recent meta-analyses on the placebo and nocebo effects in MD research highlight the influence of these potential confounders,^11^^,^^26^ underscoring the importance of performing a pivotal double-blind trial to properly assess the effectiveness of EDB.

The present trial, using a double-blind design, does not confirm a specific benefit of EDB over ESD. This finding aligns with concerns that non-specific effects, including placebo responses and expectation bias, may contribute substantially to reported improvement in Ménière’s disease. The observed association between baseline patient expectations and clinical outcome further supports this interpretation.

In addition to non-specific treatment effects, the natural course of Ménière’s disease needs to be considered. In a previously reported wait-list cohort from our group with identical inclusion criteria and outcome definitions, 64% of patients became free from vertigo attacks within 2 years without surgical intervention.^27^ Similar rates of spontaneous improvement have been reported in other observational cohorts.^28^, ^29^, ^30^, ^31^, ^32^, ^33^, ^34^ These findings suggest that a substantial proportion of patients may experience disease remission over time independent of surgical treatment. Consequently, the improvement observed in both study arms may reflect a combination of natural disease evolution and non-specific treatment effects rather than a procedure-specific benefit.

The choice of an appropriate control intervention required balancing methodological rigour with ethical feasibility. A non-surgical control arm was considered but rejected because it would not have allowed maintenance of blinding and would therefore have increased susceptibility to expectation-related bias, which is particularly relevant for Ménière’s disease.

ESD was chosen because it is a surgical procedure very similar to EDB, with the only difference being the clip placement. Although ESD has previously been considered by some as approximating a placebo procedure, the evidence remains inconclusive, and its ongoing clinical use reflects continued uncertainty about its true efficacy.^35^, ^36^, ^37^ Altogether, the chosen comparator (ESD) represented a pragmatic compromise, allowing preservation of blinding while maintaining close procedural similarity.

In addition to standard visit-based assessments, the DizzyQuest app was used to enable real-time reporting of vertigo attacks, with the aim of minimising recall bias. Visit-based assessment constituted the prespecified primary endpoint, whereas app-based data were considered exploratory.

While visit-based analyses showed no difference between the groups, app-based reporting suggested a higher proportion of attack-free patients in the EDB group. However, the clinical relevance of this finding is uncertain, as the absolute number of days with attacks was low in both groups, especially in light of the large number of days *without* attacks. The difference in days with attacks was not statistically significant, and no differences were observed in attack severity or patient-reported outcomes such as quality of life. Furthermore, several outliers in the ESD group influenced the results. Taken together, these findings suggest that while real-time reporting may reduce bias, it does not alter the overall conclusion that EDB does not have a clinically meaningful benefit over ESD.

Given the absence of demonstrable superiority, the risk profile of EDB is particularly relevant when considering its role in clinical practice. Serious adverse events occurred in 4% of cases. Although endolymphatic sac surgery is generally considered safe, this rate is higher than reported in previous literature.

Overall, the balance of benefit and harm does not support a meaningful advantage of EDB over ESD. In clinical practice, this underscores the importance of careful patient counselling, with emphasis on the uncertain efficacy of surgical intervention and the potential for spontaneous improvement over time.

The main strength of this trial lies in its double-blind design, which is particularly important in a condition with substantial placebo effects. Another strength is the presence of the same experienced surgeon at every procedure, to exclude variation in technique or experience among surgeons. Furthermore, randomisation was stratified by sex and disease duration to control for potential confounding.

Several limitations should be acknowledged. The follow-up period was 12 months, although current guidelines recommend two years of follow-up.^38^ In addition, the absence of a non-surgical placebo or pure sham control limits the ability to quantify absolute treatment effects beyond non-specific effects. Finally, potential biological heterogeneity within Ménière’s disease, including anatomical and genetic variability,^39^ was not explicitly addressed and may contribute to variation in treatment response. However, no validated or universally accepted framework currently exists to enable reliable stratification of such subgroups in clinical trials. Nevertheless, the randomised design of this trial is expected to minimise the risk of systematic confounding of such potential subgroups across treatment arms.

These findings have important implications for the design of future studies in Ménière’s disease. Given the influence of expectation effects, rigorous control of bias is essential. While sham-controlled surgical trials would provide the most definite evidence, their implementation is often limited by ethical and practical considerations.

Alternative strategies may include a non-surgical control arm when feasible, systematic measurement of patient expectations and integrating objective and continuously reported outcomes to reduce recall bias. In addition, future research into biological or imaging-based phenotyping may enable more refined stratification, but such approaches are not yet sufficiently validated for clinical trial application.

In conclusion, this trial does not demonstrate a benefit of endolymphatic duct blockage over endolymphatic sac decompression. The findings suggest that observed improvements may reflect a combination of non-specific treatment effects and the natural course of MD rather than a specific surgical mechanism. Real-time reporting highlights potential limitations of conventional recall-based assessment but does not change overall interpretation.

Taken together, the findings do not support implementation of EDB in routine clinical practice. Management of Ménière’s disease should prioritise patient education, shared decision-making, and realistic counselling regarding the uncertain benefits of surgical intervention and the likelihood of spontaneous improvement over time.

## Contributors

AS, PB, JK, and HB contributed to conceptualisation and methodology of the study. AS was responsible for project administration, coordinated the trial, curated the data, and drafted the original manuscript. RB, TP, YS, SW, AH, and JW contributed to investigation and data collection as local investigators. SHa contributed to methodology and radiological assessment. SHo and MM contributed to methodology, physiotherapy assessments, and investigation. SB performed the formal statistical analysis. HB performed the surgical procedures and supervised the study.

All authors contributed to data interpretation and critically revised the manuscript for important intellectual content. All authors approved the final version of the manuscript.

AS, SB, and HB directly accessed and verified the underlying data reported in the manuscript and had full access to all the data in the study. AS and HB had final responsibility for the decision to submit the manuscript for publication.

## Data sharing statement

Deidentified participant data, including individual participant data, statistical code, and a data dictionary defining each field in the set of this study are made available upon request. Other documents such as study protocol including statistical analysis plan will also be available. Data will only be made available on request and for research purposes and only after approval of a research proposal. A data sharing agreement will have to be signed by both parties before data are made available.

## Use of generative AI

ChatGPT (OpenAI) was used for language editing, including grammar correction and improvement of clarity, flow, and English writing style. It was not used for study conceptualization, data analysis, interpretation, or scientific decision-making. All content was reviewed and approved by the authors.

## Declaration of interests

AS: employed as a resident in training at Leiden University Medical Center since September 2023.

RB: the author’s institution received grants or contracts from ZonMw, Stichting Weijerhorst, Stichting Care for Quality of Life, Stichting Heinsius Houbolt Fonds, MEDEL, and Elitac outside the submitted work. The author received honoraria from Abbott for lectures and presentations. The author is unpaid chair of the educational committee of the Bárány Society. The author reports no other conflicts of interest.

All other authors declare no competing interests.

## Appendix A Supplementary data

Supplementary Tables S1–S3

Trial protocol
