# Supplementary material for: Endolymphatic duct blockage for Ménière’s disease: a double-blind, randomised controlled trial
Source: Lancet Reg Health Eur. 2026 Jul 6;68:101765. doi: 10.1016/j.lanepe.2026.101765 (PMC13355790; doi:10.1016/j.lanepe.2026.101765)
Supplement: Supplementary Tables S1–S3 [file mmc1.pdf]

Supplementary material

Table of contents

**Supplementary material**	**Description**	**Page**
Supplementary table 1	Full inclusion and exclusion criteria	2
Supplementary table 2	Details of all follow-up visits	3
Supplementary table 3	Descriptive statistics for all repeated outcome measures by treatment group	4-5

**Inclusion criteria**	
	Definite unilateral MD according to diagnostic criteria of the Bárány Society(16)
	More than 3 patient reported attacks in the 6 months prior to inclusion and at least 1 attack in the 2 months prior to inclusion
	Age ≥ 18 years at the start of the trial
	Not responding to a sufficient extent to conservative medical treatment including at least two sessions of at least one intra-tympanic injection (IT) each with corticosteroids (dexamethasone, methylprednisolone, triamcinolonacetonide)[1]
	Dutch health care insurance
**Exclusion criteria**	
	Severe disability (e.g. neurological, orthopedic, cardiovascular) according to the investigator, pregnancy or serious concurrent illness that might interfere with surgery or follow-up.
	Active additional neuro-otologic disorders that may mimic MD (e.g. vestibular migraine (VM), recurrent vestibulopathy, phobic postural vertigo, vertebro-basilar TIAs, acoustic neuroma, congenital disorders, enlarged vestibular aquaduct (EVA)-like or genetic disorders (like DFNA9), cervicogenic dizziness), based on the complete clinical record.
	Previous ear surgery for MD (IT injection is not an exclusion criterion)
	Language difficulties
	Active otitis media (with or without effusion)
	Unable or unwilling to use DizzyQuest App
	Unable to undergo MRI (such as gadolinium allergy, claustrophobia, implanted non-MRI compatible device of material, BMI)
	Deafness of the contralateral ear

Supplementary Table 1.

**Preoperative visit**	
	ENT-surgeon
	Physiotherapy
	(v)HIT
	PTA
	MRI scan
	CT scan
	ENG
	Questionnaires: HADS, DHI, THI, FLS, EQ-5D VAS, SF36, NPQ, VADL, VAP, assessment of expectations of patients
**1 week follow-up**	
	ENT: wound inspection
	Physiotherapy
**3 months follow-up**	
	ENT, including (video)HIT
	Physiotherapy
	PTA
	MRI scan
	Questionnaires: DHI, THI, FLS, EQ-5D VAS, SF36, iMCQ, iPCQ
**6 months follow-up**	
	ENT, including (video)HIT
	Physiotherapy
	PTA
	Questionnaires: DHI, THI, FLS, EQ-5D VAS, SF36, iMCQ, iPCQ
**12 months follow-up**	
	ENT, including (video)HIT
	Physiotherapy
	PTA
	MRI
	ENG
	Questionnaires: DHI, THI, FLS, EQ-5D VAS, SF36, iMCQ, iPCQ, VADL, VAP

Supplementary Table 2.

		EDB			ESD		
**Outcome**	**Time**	**Sample size**	**Mean**	**SD**	**Sample size**	**Mean**	**SD**
**FLS**	Baseline	37	4·2	0·9	36	3·9	1·1
	3 months	35	3·1	1·4	35	3·1	1·4
	6 months	36	3·0	1·6	33	3·0	1·4
	12 months	38	2·7	1·6	33	3·1	1·1
**SF-36 physical**	Baseline	36	40·7	7·8	35	43·8	7·4
	3 months	34	44·2	7·9	35	44·7	7·8
	6 months	36	44·6	9·2	33	44·3	7·7
	12 months	38	46·0	8·7	33	45·6	8·0
**SF-36 mental**	Baseline	36	40·6	6·9	35	41·0	6·6
	3 months	34	42·4	6·1	35	42·7	6·6
	6 months	36	42·7	6·9	33	43·0	5·5
	12 months	38	43·1	6·8	33	43·8	5·5
**EQ-5D**	Baseline	39	0·86	0·11	36	0·87	0·11
	3 months	39	0·89	0·12	36	0·89	0·09
	6 months	39	0·87	0·15	36	0·90	0·10
	12 months	39	0·87	0·15	36	0·90	0·09
**VAS**	Baseline	37	61·2	17·1	36	66·4	18·5
	3 months	35	69·8	17·2	35	71·4	20·1
	6 months	36	70·4	21·8	33	68·8	23·0
	12 months	38	69·9	25·5	33	70·1	20·5
**DHI**	Baseline	37	61·7	18·4	36	50·7	18·2
	3 months	35	36·9	24·9	35	37·4	22·2
	6 months	36	35·2	28·5	33	37·6	23·9
	12 months	38	33·2	26·5	33	36·2	22·8
**THI**	Baseline	37	45·0	24·9	36	32·8	22·4
	3 months	35	32·2	23·5	35	28·3	21·2
	6 months	36	35·4	26·9	33	29·0	21·9

	12 months	38	34·0	27·7	33	26·6	19·2
**Hearing (PTA, affected ear)**	Baseline	39	54·5	17·4	36	50·6	14·0
	3 months	37	48·8	25·8	36	55·2	20·2
	6 months	38	48·6	22·7	35	52·3	18·2
	12 months	36	48·0	22·5	36	51·4	18·3
**Hearing (PTA, unaffected ear)**	Baseline	39	18·6	9·6	36	17·2	13·7
	3 months	37	17·7	9·7	36	18·8	13·9
	6 months	38	18·7	10·3	35	17·4	14·0
	12 months	36	18·7	9·1	36	18·7	13·7
**Speech discrimination (SDS, affected ear)**	Baseline	35	76·8	27·7	33	75·9	24·8
	3 months	38	78·3	30·0	27	71·0	35·7
	6 months	39	77·4	31·7	26	71·1	27·8
	12 months	39	80·9	27·6	27	65·7	32·3
**Speech discrimination (SDS, unaffected ear)**	Baseline	35	99·1	4·3	32	98·0	8·0
	3 months	38	99·5	1·4	26	96·6	8·6
	6 months	39	100·0	0	25	95·4	11·2
	12 months	39	100·0	0	26	96·3	7·2
**Vestibular function (affected ear)**	Baseline	36	10·7	8·5	34	9·2	6·9
	12 months	32	8·2	6·4	29	9·7	7·0
**Vestibular function (unaffected ear)**	Baseline	36	14·4	11·5	34	15·0	13·3
	12 months	32	13·9	8·7	30	18·8	12·5

Supplementary Table 3.
