# Supplementary material for: Endolymphatic duct blockage for Ménière’s disease: a double-blind, randomised controlled trial
Source: Lancet Reg Health Eur. 2026 Jul 6;68:101765. doi: 10.1016/j.lanepe.2026.101765 (PMC13355790; doi:10.1016/j.lanepe.2026.101765)
Supplement: Trial protocol [file mmc2.pdf]

RESEARCH PROTOCOL

EDB trial

May 2023

The effectiveness of endolymphatic duct blockage versus endolymphatic sac decompression in patients with intractable Ménière’s disease

PROTOCOL TITLE *'The effectiveness of endolymphatic duct blockage versus endolymphatic sac decompression in patients with intractable Ménière's disease'*

**Protocol ID**	RCT KNO EDB Trial 2020
**Short title**	EDB Trial
**Version**	10
**Date**	08-05-2023
**Project leader**	H. Blom, MD, PhD<br>ENT-surgeon<br>Department of Otorhinolaryngology<br>HagaZiekenhuis, The Hague, the Netherlands<br>Tel: + 31 (0) 638828888<br>h.blom@hagaziekenhuis.nl
**Coordinating investigator**	A.A. Schenck, MD<br>PhD-student<br>Department of Otorhinolaryngology<br>HagaZiekenhuis, The Hague, the Netherlands<br>Tel: +31 6 20280814<br>j.schenck@hagaziekenhuis.nl
**Principal investigators**	J.M. Kruyt, MD<br>ENT-surgeon<br>Department of Otorhinolaryngology<br>Bergman Clinics, Rijswijk, the Netherlands<br>Tel: + 31 88 979 24 45<br>k.kruyt@bergmanclinics.nl<br><br>P.P.G. van Benthem, MD, PhD<br>Professor of Otorhinolaryngology<br>Chair Department of ORL-HNS<br>Department of Otorhinolaryngology<br>Leiden University Medical Centre, Leiden, the Netherlands<br>+31 71 526 2434<br>p.van.benthem@lumc.nl
**Other investigators**	Apeldoorns Duizeligheidscentrum, Gelre Ziekenhuis:

	Dr. S.M. Winters<br><br>Beatrix Ziekenhuis, Gorinchem: dr. Y.E. Smulders<br><br>Maastricht UMC+: dr. R. van der Berg<br><br>Medisch Centrum Leeuwarden: dr. T. Peters<br>Wilhelmina Ziekenhuis Assen: dr. A.F. Holm<br><br>Additional (contact) information can be found in the appendix (Appendix 1).
**Sponsor (in Dutch: verrichter/opdrachtgever)**	HagaZiekenhuis, The Hague
**Subsidising party**	Veelbelovende zorg
**Independent expert (s)**	M. Van der Schroeff, PhD<br><br>ENT-surgeon<br><br>Erasmus Medical Centre, Rotterdam
**Laboratory sites**	NA
**Pharmacy**	NA

PROTOCOL SIGNATURE SHEET

**Name**	**Signature**	**Date**
**Head of ENT Department LUMC:**<br>P.P.G. van Benthem		
**Project leader**		
H.M. Blom		
**Coordinating Investigator**		
A.A. Schenck		
**Principal investigators**		
J.M. Kruyt		
**Other investigators**		
R. van de Berg		
A.F. Holm		
Y.E. Smulders		

T. Peters	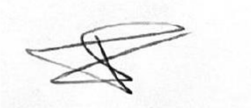	
S.M. Winters	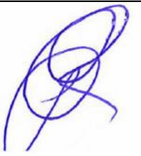	

TABLE OF CONTENTS

1. INTRODUCTION AND RATIONALE 13
2. OBJECTIVES 19
3. STUDY DESIGN 20
4. STUDY POPULATION 22
4.1 Population (base) 22
4.2 Inclusion criteria 22
4.3 Exclusion criteria 23
4.4 Sample size calculation 24
4.5 Numbers needed to treat 25
5. TREATMENT OF SUBJECTS 26
5.1 Investigational treatment 26
5.2 Use of co-intervention 27
5.3 Escape medication 27
6. INVESTIGATIONAL PRODUCT 29
7. NON-INVESTIGATIONAL PRODUCT 29
8. METHODS 30
8.1 Study parameters/endpoints 30
8.1.1 Main study parameter 30
8.1.2 Secondary study parameters 30
8.2 Randomisation, blinding and treatment allocation 34
8.2.1 Process of randomisation 34
8.2.2 Blinding of randomisation and follow up 35
8.2.3 Emergency unblinding 35
8.3 Study procedures 35
8.3.1 Preoperative procedures (T-1) 35
8.3.2 Surgical procedure (T=0) 38
8.3.3 Postoperative procedures 38
8.3.4 Flow chart 40
8.4 Withdrawal of individual subjects 41
8.5 Replacement of individual subjects after withdrawal 41
8.6 Follow-up of subjects withdrawn from treatment 41
8.7 Premature termination of the study 41
9. SAFETY REPORTING 42
9.1 Temporary halt for reasons of subject safety 42
9.2 AEs, SAEs and SUSARs 42
9.2.1 Adverse events (AEs) 42
9.2.2 Serious adverse events (SAEs) 43
9.2.3 Suspected unexpected serious adverse reactions (SUSARs) 44
9.3 Annual safety report 44
9.4 Follow-up of adverse events 44
9.5 Data Safety Monitoring Board (DSMB) 44

10. STATISTICAL ANALYSIS 46
10.1 Primary study parameter 46
10.1.1 Imputation of App-recorded data 46
10.1.2 Computation of outcome 47
10.2 Secondary study parameters 47
10.2.1 Missing data 47
10.2.2 Computation of outcome 47
10.3 Other study parameters 47
10.4 Interim analysis 47
11. ETHICAL CONSIDERATIONS 48
11.1 Regulation statement 48
11.2 Recruitment and consent 48
11.3 Objection by minors or incapacitated subject 48
11.4 Benefits and risks assessment, group relatedness 48
11.5 Compensation for injury 49
11.6 Incentives 49
11.7 Other ethical considerations 50
11.7.1 Support of other relevant communities 50
12. ADMINISTRATIVE ASPECTS, MONITORING AND PUBLICATION 51
12.1 Handling and storage of data and documents 51
12.2 Monitoring and Quality Assurance 53
12.3 Amendments 53
12.4 Annual progress report 53
12.5 Temporary halt and (prematurely) end of study report 53
12.6 Public disclosure and publication policy 54
12.6.1 Data analysis and release of results 54
12.6.2 Review process 55
12.6.3 Close-out procedures 55
13. STRUCTURED RISK ANALYSIS 56
14. REFERENCES 57
15. APPENDICES 63
15.1 Appendix 1 - Investigator names and contact data 63
15.2 Appendix 2 – Extensive flowchart patient visits 68
15.3 Appendix 3 – Full vestibular rehabilitation therapy 69
15.4 Appendix 4 – IT injection procedure 74
15.5 Appendix 5 – Radiology protocol 75
15.6 Appendix 6 – Monitoringplan HagaZiekenhuis 77

LIST OF ABBREVIATIONS AND RELEVANT DEFINITIONS

AAO-HNS	American Academy of Otolaryngology-Head and Neck Surgery
ABR	General Assessment and Registration form (ABR form), the application form that is required for submission to the accredited Ethics Committee; in Dutch: Algemeen Beoordelings- en Registratieformulier (ABR-formulier)
AE	Adverse Event
BIA	Budget Impact Analysis
BPPV	Benign paroxysmal positional vertigo
CATS	Caffeine, alcohol, theophylline, salt
CCMO	Central Committee on Research Involving Human Subjects; in Dutch: Centrale Commissie Mensgebonden Onderzoek
CEA	Cost Effectiveness Analysis
CSF	Cerebrospinal fluid
CT	Computed Tomography
CV	Curriculum Vitae
dB	Decibels
DGI	Dynamic Gait Index
DHI	Dizziness Handicap Inventory
DSMB	Data Safety Monitoring Board
DVA	Dynamic visual acuity
DVAT-NI	Dynamic visual acuity test – non instrumented
ED	Endolymphatic duct
EDB	Endolymphatic Duct Blockage
EDC	Electronic data capture
ENT	Ear, nose, throat
EQ-5D	EuroQoL 5 dimensional
ES	Endolymphatic Sac
ESD	Endolymphatic Sac Decompression
EVA	Enlarged vestibular aquaduct
FLS	Functional Level Scale
GCP	Good Clinical Practice
GDPR	General Data Protection Regulation; in Dutch: Algemene Verordening Gegevensbescherming (AVG)
HADS	Hospital Anxiety and Depression Score
HT	Hearing threshold

IC	Initiating Centre
iMCQ	iMTA Medical Costs Questionnaire
iPCQ	iMTA Productivity Costs Questionnaire
IT	Intratympanic
LFI	Low Fletcher Index
METC	Medical research ethics committee (MREC); in Dutch: medisch-ethische toetsingscommissie (METC)
MD	Ménière's disease
MRI	Magnetic Resonance Imaging
NNT	Numbers needed to treat
OR	Operation room
PC	Participating Centre
PROM	Patient reported outcome measures
PSCC	Posterior semi-circular canal
PTA	Pure tone, speech audiometry
QoL	Quality of life
RCT	Randomized controlled trial
(S)AE	(Serious) Adverse Event
SF-36	Short Form Survey 36
Sponsor	The sponsor is the party that commissions the organisation or performance of the research, for example a pharmaceutical company, academic hospital, scientific organisation or investigator. A party that provides funding for a study but does not commission it is not regarded as the sponsor, but referred to as a subsidising party.
SPSS	Statistical Package for the Social Sciences
THI	Tinnitus Handicap Inventory
TIA	Transient ischemic attack
UAVG	Dutch Act on Implementation of the General Data Protection Regulation; in Dutch: Uitvoeringswet AVG
VADL	Vestibular Disorders Activities of Daily Living Scale
VAP	Vestibular Activitities and Participation Measure
VAS	Visual Analogue Score
vHIT	Video head impulse test(ing)
VM	Vestibular migraine
VOR	Vestibulo-ocular reflex

WMO	Medical Research Involving Human Subjects Act; in Dutch: Wet Medisch-wetenschappelijk Onderzoek met Mensen
WR	Word recognition
ZIN	Zorginstituut Nederland

SUMMARY

Rationale: Ménière's disease (MD) is an incapacitating disease in which recurrent attacks of vertigo are accompanied by hearing loss, tinnitus and/or aural fullness. A population of 60-100 per 100.000 patients in the Netherlands is severely impaired (low quality of life) by the disease. Current treatments have either proven to be ineffective (Betahistin), destroy the labyrinth function (intratympanic gentamicin and ablative surgery) or only provide a temporary solution (intratympanic corticosteroid injections). In many countries, surgery on the endolymphatic sac, such as decompression, shunting or drainage, is part of standard care for Meniere's disease, but not in the Netherlands and Scandinavian countries. Recently, a new, surgical technique has been published by Saliba et al. This technique, referred to as Endolymphatic Duct Blockage (EDB), involves blocking the connection of the endolymphatic sac with the inner ear by clipping the endolymphatic duct (ED). A paradigm shift for the pathophysiological model of Ménière's disease underlies this new treatment. Patients with MD have a hydrops of the endolymphatic system as can be demonstrated by MRI (3 Tesla). Until now, it is believed that the surplus of endolymph causing the hydrops originates in the cochlea and the vestibular organ. However, Saliba et al. state that the surplus of endolymph originates in the endolymphatic sac (ES) and that Ménière's disease originates from the ES as well. Saliba et al. report very favourable results of EDB, but their study was methodologically flawed, as it was not blinded. Therefore, we feel obliged to perform a so-called pivotal trial to establish whether EDB is more effective than endolymphatic sac decompression for controlling vertigo in patients suffering from MD.

Objective: The objective of this study is to evaluate the effectiveness of surgical clipping of the ED in participants with Ménière's disease, as compared to the decompression procedure where the duct is not clipped. We expect that the number of patients free of vertigo attacks at 12-months postoperative will be higher in the EDB group than in the decompression group. In addition, we hypothesize that there will be less hearing loss, tinnitus, loss of vestibular function and hydrops, and an increase in quality of life (QoL) in participants in the EDB group.

Study design: This is a double-blinded, randomized controlled trial. Total duration of the study is 4 years. Minimal study duration per participant is 1-year post surgery. All of the operations will take place in participating centres within a fixed period of 2 years after the first patient is operated. Surgery will be performed by two surgeons simultaneously. One of the surgeons will leave the operating room before the randomisation. This surgeon is blinded to the treatment and will take care of the follow-up. Randomization will be 1:1 stratified for gender and duration of MD (recent onset versus mature MD).

Study population: The study will include 74 participants suffering from MD who meet the diagnostic criteria as recently revised in 2015. These patients have not responded to more conservative treatment modalities.
Intervention: Participants from both study groups will undergo mastoidectomy with identification of the ED. In the EDB group, the ED will be clipped and in the decompression group, it will not be clipped. All participants receive vestibular rehabilitation after surgery. Follow up visits will take place at 1 week, 3 months, 6 months and 12 months after surgery.
Main study endpoint: Proportion of patients who are free of vertigo spells at 12 months post-operative.
Nature and extent of the burden and risks associated with participation, benefit and group relatedness: All participants will have ear surgery, repeated testing and questionnaires and report daily in the DizzyQuest app. Usual risks of surgery apply. We expect patients in both groups to benefit from participation, either because of the effect of the surgical interventions, or because of the considerable placebo effect.
An interim analysis will be performed after 21 participants have undergone surgery to assess surgery related risks and to end the trial if safety of participants cannot be guaranteed.

1. INTRODUCTION AND RATIONALE

- Ménière's disease

Ménière's disease (MD) is an incapacitating disease of recurrent vertigo attacks, accompanied by hearing loss, tinnitus and/or aural fullness. Between the attacks of vertigo intervals of days, weeks or even months may occur (Lopez 2015, Pullens 2013). The natural course of MD has been studied and it has been found that the attacks of vertigo become less severe and disappear after two years in 60% and after eight years in 80% of patients (Portmann 1980; Silverstein 1989, Perez 2008, Van Esch 2016). In the end phase of the disease patients without vertigo attacks may still suffer from lasting hearing loss and tinnitus and chronic instability caused by hypofunction of the labyrinth.
Significant comorbidity is seen in patients with Ménière's disease. The most important comorbidities that may also cause dizziness are anxiety disorders and BPPV (van Esch, 2016).

- Pathophysiology

MD is an idiopathic disease associated with endolymphatic hydrops in the inner ear (Merchant, 2005). Visualization of the hydrops became possible with the introduction of delayed post-contrast high resolution MR imaging (Nakashima 2007; Naganawana, 2014; Baráth, 2014). Moreover, recent publications underline the relevance of the signal intensity of the perilymphatic fluid (Bernaerts 2019, Shi 2018, Steekelenburg 2020). This signal intensity is a surrogate marker for the permeability of the blood-perilymph barrier, and may therefore reflect acute inflammation or recent activity related to a vertigo attack.

- Epidemiology

Few articles have been published on the epidemiology of MD. Great variation exists in the published reports of the prevalence of MD, ranging from 34.5 cases per 100,000 population in Japan (Shojaku 2005) to 46 cases per 100,000 population in Sweden (Stahle 1978). In the United States of America, reported prevalence is much higher: Alexander (Alexander 2010) reports 190 patients per 100,000, Waldislavosky-Waserman (Wladislavosky-Waserman 1984) reports a prevalence of 218 per 100.000. The difference in prevalence might be due to the wide variations in definitions of MD.
In the Netherlands, a population of 60-100 per 100,000 patients is severely impaired (defined as low quality of live) by the disease (Mateijsen, 2001). There seems to be a slight female preponderance, with up to 1.3 times more women affected than men. The disease is more common in adults in their fourth and fifth decade of life (Kotimaki 1999, Perez 2008, Van Esch 2016).

- Current treatment options and guidelines

The treatment of MD both in primary and secondary care setting is focused on the reduction of the frequency and intensity of vertigo attacks. Current treatments have either proven to be ineffective (Betahistin;Adrion 2016), only have a temporary effect (intratympanic dexamethasone injections; McRackan 2014, or methylprednisolone; Patel 2016), or destroy the labyrinth function (intratympanic gentamicin, labyrinthectomy, selective neurectomy;Pullens 2013; Harner 2001, Sennaroglu 2001). Surgical destruction of the labyrinth reduces the episodes of attacks but causes loss of balance as well, due to one dysfunctional labyrinth. Moreover, permanent hearing loss is reported after this treatment.

Recently, an international guideline for the diagnostic work-up and treatment of MD was published (Basura 2020). It recommends step-up treatment, starting with education of patients and discussing diuretics/betahistine. IT corticosteroids are considered optional if patients do not respond to more conservative therapy. A last non-ablative option that can be considered, is endolymphatic sac decompression, however, results from this type of surgery are discordant. If there is no response to non-ablative treatments, treatment with intratympanic gentamicin is recommended, and if the disease remains unmanageable and the patient has nonusable hearing, labyrinthectomy is advised. Patients should also be referred for vestibular rehabilitation therapy in case of chronic balance problems, and clinicians should counsel patients with hearing problems about hearing assistive technology.

In the Netherlands, there are currently no specific guidelines for the treatment of MD. The guideline ‘Dizziness in Elderly’ of the Dutch ENT Society (‘Duizeligheid bij ouderen’, NVNKO, 2015) advises to refer the patients to a specialized ENT surgeon. The guidelines ‘Dizziness’ of the Dutch General Practitioner Society (Bouma 2017) for general practitioners also advises to refer the patient to an ENT surgeon, but only in case of rapid progressive hearing loss or additional complaints such as instability, head ache of persisting tinnitus. There is no guideline from the Dutch ENT society regarding Meniere’s disease.

- Endolymphatic sac surgery

Other surgical treatment techniques target the endolymphatic sac (ES). The advantage of these procedures is that they are non-destructive and do therefore not affect the cochlear and vestibular function, thereby preserving hearing and balance. These procedure involve decompression, shunting or drainage of the ES.

Endolymphatic sac decompression (ESD) consists of a mastoidectomy and, after identification of the endolymphatic sac, wide decompression of this structure (Sennaroglu,

2001). ESD has few surgical complications in comparison with the ablative surgery mentioned above. There is no consensus on the effect of decompression. Convert et al (Convert 2006) report improved quality of life after decompression in a case study of 90 subjects after a follow up period of 57.5 months on average.

Drainage or shunting of the endolymphatic sac (ESS) involves identification of the ES, followed by incision of the sac. A shunt is then placed, enabling drainage of the endolymph. There are several studies that were directed to investigate the effectiveness of ESS (Bretlau 1989, Thomsen 1998, Brinson 2007). Bretlau and Thomsen compared ESS to a sham operation; no differences between the groups was observed. Brinson compared ESS to ESD performed on 88 and 108 patients, respectively. He concluded that both procedures are effective

Multiple histological studies refute the rationale of endolymphatic sac surgery. Firstly, Chung et al (2011), performed a histopathological study 15 patients who had undergone ESS. If the endolymphatic sac does indeed have a function in resorption of the endolymph but does so inadequately, ESS and especially ESD would allow expansion of these structures and would therefore diminish hydrops. However, diffuse hydrops on temporal bone was seen in the cochlea, the saccule, the utricle, and the ampulla after ESD. The authors conclude that ESD does not relieve hydrops in patients with Ménière’s disease.
In addition, if the ES was responsible for endolymph resorption, an increase of hydrops can be expected after amputation of the ES. However, Linthicum et al. (2011) reported a case in which removal of the ES did not lead to an increase of hydrops, as seen on temporal bone histopathology. In the assessed samples, Reissner’s membrane was attached to the spiral ligament in a normal way, without any evidence of hydrops in the cochlea. In conclusion, the role of the ES is not merely absorption of the endolymph and therefore, providing more space to allow dilatation is not the solution for the observed hydrops.

The success rates of these surgical interventions vary between 30-95% (Sennaroglu 2001; Huang 1991; Silverstein 1989, Durland 2005, Pullens 2013; Convert 2006). It should be noted that the natural course of MD is also favourable, and it cannot be determined to what extent this outcome is due to the surgical intervention. Moreover, the placebo effect may play a major role in the relief of complaints, as 70% of MD patients in all groups (all surgical interventions as well as the control groups) experienced some relief of complaints. This either implicates a beneficial effect of any surgical intervention or of any intervention, be it surgical or non-surgical. This was earlier suggested by Thomsen (Thomsen 1981).

The newly published international guideline makes no recommendation regarding the use of endolymphatic sac surgery, ‘due to its uncertain benefit and discordant results when comparing small controlled studies and larger and more numerous uncontrolled studies’ (Basura 2020). However, the guideline acknowledges that is utilized by clinicians.
Data from America, Asia and Europe on decompression have been published (Xu 2020, Kitahara 2014, Bojrab 2018, Convert 2006). However, in Scandinavian countries and the Netherlands, the general view is that these surgeries have never proven to be more effective than placebo surgery, and are therefore not performed as part of usual care.

- Endolymphatic duct blockage

Recently, a new surgical intervention has been studied by Saliba et al. (Saliba 2015). A paradigm shift for the pathofysiological model of MD underlies this new treatment. Until now it is believed that the disease is caused by a surplus of endolymph originating in the inner ear, caused by a disequilibrium in the production of endolymph in the inner ear and its resorption in the endolymphatic sac (Merchant, 2005, Semaan, 2010, Salt, 2010). However, Saliba et al. state that the organic substrate of the disease - the surplus of endolymph causing the hydrops – also originates in the endolymphatic sac (ES).

The idea that the surplus of endolymph originates in the ES, is supported by two studies that suggest that the ES has secretory functions as well, rather than merely a function in absorption. In a study of the subcellular structure of the endolymphatic sac in guinea pigs by Takumida et al (1987), the presence of dark cells in the endolymphatic sac was shown. These cells have a secretory role. Moreover, a study performed by Friis (2013) on Lewis rats showed hyperactivity of the cells of the endolymphatic sac, leading to an increase of endolymph secretion.
In conclusion, histological evidence that the ES is –at least in part- responsible for the endolymph surplus.

Based on these findings, Saliba’s hypothesis is that in Ménière’s disease, there is imbalance in the fluid homeostasis of the endolymph at the level of the endolymphatic sac. He argues there is an increased secretion outweighing a decreased absorption in the ES, leading to increased pressure in the inner ear. Thus, by blocking the endolymphatic duct, Saliba aims to decrease the volume of endolymph in the inner ear, thereby alleviating the symptoms of Ménière’s disease.

This operation, referred to as the Endolymphatic Duct Blockage (EDB), involves placing a clip on the endolymphatic duct to separate the endolymphatic sac from the vestibulum and

the rest of the inner ear. Blockage of the endolymphatic duct impedes accumulation of endolymph in the inner ear, and therefore of vertigo attacks. Benefits of this technique are its permanent nature and the fact that it does not destroy the labyrinth or inner ear function.

Saliba (Saliba 2015) has performed a randomized trial to study the effect of EDB. The trial compared EDB to ESD and was conducted prospectively and non-blinded. There was no comparison to a group of patients receiving placebo treatment, for instance a sham operation. The results have been published in 2015 (Saliba 2015) and show that 34 of 35 treated patients were free of vertigo attacks after EDB surgery. It is interesting to note that the efficacy for the absence of vertigo attacks following ESD was only reported to be about 40% in Saliba's trial (Saliba 2015). In earlier studies by Bretlau and later Thomsen, percentages for both ESD and sham operations were reported to be as high as 70%. Possibly, this can be explained by the open character of the Saliba study, causing patients to experience the 'nocebo-effect', caused by feeling like they have not been treated because they did not have the EDB surgery (but the ESD instead). The fact that Saliba et al. did not assess outcomes in a double-blinded way is a fatal flaw in methodology given the high placebo effect of interventions in Ménière disease. Moreover, randomisation was not stratified and there is a risk of recall bias, as it is not described how vertigo bouts are recorded. Lastly, all participants were asked to follow the CATS (caffeine, alcohol, theophylline and salt restricted diet. The role of this diet is not clear.

In a more recent publication by the group of Saliba (Gabra 2016) it is reported that 43 (79%) of a group of 54 patients treated with EDB had an improved quality of life (QoL). The results of these studies indicate that EDB may have a favourable effect on both the bouts of vertigo that MD patients suffer, as on the quality of life.
It should be noted that this study was at risk for recall bias, as patients had to fill out questionnaire in retrospect.

Blom (ENT surgeon, HagaZiekenhuis, The Hague) and Kruyt (ENT surgeon, Bergman Clinics, Rijswijk) visited Saliba in Montreal to learn this surgical technique. In a pilot group of 34 patients, EDB was performed by Blom in Antwerp. The surgery was performed in Belgium because EDB is not a validated technique in the Netherlands. Therefore, performing this surgery in the Netherlands is not possible. In Belgium however, this surgery is allowed but not covered by the health insurances. Patients can have this surgery if they pay the costs themselves. In this group of 34 patients, a significant (p=.001) improvement of quality of life seen. Three of these 34 patients suffered drop attacks post-operatively, but these symptoms

were all resolved in 8 weeks. In three patients, a postsurgical cerebrospinal fluid (CSF) leakage occurred; successful surgical reintervention was performed the next day.
In addition, EDB surgery was performed on another group of 32 patients by Blom in Antwerp. No adverse events occurred in this group of patients.

According to the results of Saliba and Blom, EDB seems to be a promising surgical technique for treating MD that preserves hearing and equilibrium functions. Blom and Kruyt felt obliged to further investigate the effectiveness of the EDB in treating MD with the aim to evaluate this surgery in the Netherlands, and set up this trial in cooperation with Professor Van Benthem.
The objective of this study is to evaluate the effectiveness of EDB surgery for patients with MD, as compared to a similar intervention, endolymphatic sac decompression. We hypothesize that the number of patients without vertigo spells at 12 months follow up will be higher in the group that has undergone EDB than in the decompression group. Secondary outcomes are defined as minimally clinically significant differences in cumulative incidence of vertigo bouts, hearing, use of escape medication, co-interventions, complications of surgery, questionnaire outcomes (FLS, DHI, THI, SF-36, EQ-5D VAS, iPCQ, iMCQ), CEA, BIA, endolymphatic hydrops on MRI and physiotherapeutical outcomes. We hypothesize that the outcomes of these measures will be better in participants undergoing EDB compared to participants who have had a decompression operation.

2. OBJECTIVES

Primary objective

We will assess whether EDB surgery will result in a significant reduction in the number of patients without vertigo spells at 12 months follow up, compared to patients who undergo a decompression operation.

Secondary objectives

We will determine whether EDB surgery compared to a decompression operation during the 1-year follow up period has a beneficial effect on:

- Cumulative incidence of vertigo bouts
- Hearing
- Use of escape medication (including intratympanic injections)
- Co-interventions (ablative procedure)
- Complications of surgery
- FLS
- DHI
- THI
- SF-36
- EQ-5D VAS
- CEA
- iPCQ
- iMCQ
- BIA
- Endolymphatic hydrops and perilymph signal intensity on MRI
- Physiotherapeutical outcomes: balance, gait, visual acuity

3. STUDY DESIGN

The EDB trial is designed as a multicentre, randomized, double blind trial to compare the effectiveness of EDB surgery with decompression surgery in number of patients who are free of vertigo attacks at 12 months follow-up.

- Duration of the study

Total duration of the study is 4 years. In the first 6 months the study will be started, followed by a 2-year period for inclusion and another year for follow up. Analysis of the results will take 6 months. One year after surgery of the last included patient, randomization can be unblinded. In case of a favourable result of EDB, patients in the decompression group will be offered re-surgery to perform EDB if they still suffer attacks. This surgery will be performed within one year after deblinding. In case of a favourable outcome a trajectory for implementation in the current Dutch health care system is also foreseen. Within six months after completion of the study, Zorginstituut Nederland (ZIN) will evaluate the results and decide if the intervention should be implemented in Dutch health care.

- Justification of the design

Three initiating centres (ICs) and four participating centres (PCs) will include patients. A large number of participating centres is chosen to enable rapid inclusion of the required number of participants. As the study ends one year after surgery of the last participant included, it is important to limit the time between surgery of the first participant and last participant. This so to be able to offer the EDB to the decompression group participants who still suffer attacks within a reasonable time span in case of an outcome favourable for EDB. The multicentre design of the study facilitates fast adoption and dissemination of the surgical technique in case of a favourable outcome of EDB.

Endolymphatic sac decompression was chosen as control intervention because the procedure is very similar to EDB. When performing EDB, the same area is exposed and decompression to allow placement of the clip. Because there is no consensus about the effectiveness of decompression, research on this intervention is justified.

The placebo effect plays an important role in the treatment of MD; 70% of the patients experience improvement after any surgical intervention (Thomsen 1981, Bretlau 1989, Thomsen 1998, Pullens 2013). However, because the interventions are equal in intensity, i.e. surgical treatments. Therefore we do expect similar placebo effects in the groups.

- Flow chart

Patients will be included in one of the 7 centres that take part in this study. There will be baseline questionnaires, imaging and testing. There will be a standard preoperative screening by the anaesthetist. The surgery will take place in the centre where the patient is included and where the follow up takes place, unless the local researcher prefers to perform surgery in the HagaHospital (due to experience with the surgery).
After surgery, there will be follow up visit at 1 week, 3 months, 6 months and 12 months after surgery. Throughout this period, patients will continuously report daily functioning and vertigo attacks in the DizzyQuest app. Moreover, the patient is treated by a vestibular therapist in the first 3 months after surgery.
After one year of follow up, both patient and the follow up ENT-surgeon are deblinded. If the patient was allocated to the EDB group, a CT-scan will be performed to assess if the clip is correctly in place if the patient still suffers attacks.
If the results of this trial are in favour of EDB, patients in the decompression group who still suffer vertigo attacks will be offered EDB when the last patient has been deblinded.
An extensive flow chart, including all the questionnaires and tests that will be performed, can be found in the appendix (Appendix 2).

4. STUDY POPULATION

4.1 Population (base)

Patients with MD will be recruited in all 7 centres. These patients will typically be under treatment by one of the participating centres. Those patients meeting the inclusion/exclusion criteria will be informed by their ENT-surgeon. Below an estimate is provided per centre of the number of patients eligible for this study based on historic data.

Centre	Expected number of patients eligible for study
Beatrix Ziekenhuis, Gorinchem	2
Gelre Ziekenhuizen - Apeldoorns Duizeligheidscentrum	2
HagaZiekenhuis Den Haag	62
Leids Universitair Medisch Centrum	2
Maastricht UMC	2
Medisch Centrum Leeuwarden	1
Wilhelmina Ziekenhuis Assen	3

Based on these estimates, we expect to be able to recruit the total number of 74 patients within the 2 years in which inclusion is planned. There will not be a set number of inclusion per centre: inclusion will happen according to ‘first come first serve’ principle, which means that inclusion will be competitive.

4.2 Inclusion criteria

In order to be eligible to participate in this study, a patient must meet all of the following criteria:

- Definite unilateral MD according to diagnostic criteria of the Bárány Society (Lopez-Escamez, 2016)
- More than 3 patient reported attacks in the 6 months prior to inclusion and at least 1 attack in the 2 months prior to inclusion
- Age ≥ 18 years at the start of the trial
- Non responding to a sufficient extent to conservative medical treatment including at least two sessions of at least one intra-tympanic injection (IT) each with corticosteroids (dexamethasone, methylprednisolone, triamcinolonacetonide)
- Dutch health care insurance

Definite MD according to Bárány Society:

- Two or more spontaneous episodes of vertigo, each lasting 20 minutes to 12 hours,

AND

- Audiometrically documented low- to medium-frequency sensorineural hearing loss* in one ear, defining the affected ear on at least one occasion before, during or after one of the episodes of vertigo,

AND

- Fluctuating aural symptoms (hearing, tinnitus, or fullness) in affected ear (not better accounted for by another vestibular diagnosis).

*Low frequency sensorineural hearing loss is defined as increases in pure tone thresholds for bone-conducted sound that are higher (i.e. worse) in the affected ear than the contralateral ear by at least 30 dB HL at each of two contiguous frequencies below 2000 Hz

4.3 Exclusion criteria

A potential subject who meets any of the following criteria will be excluded from participation in this study:

- Severe disability (e.g. neurological, orthopedic, cardiovascular) according to the investigator, pregnancy or serious concurrent illness that might interfere with surgery or follow-up.
- Active additional neuro-otologic disorders that may mimic MD (e.g. vestibular migraine (VM), recurrent vestibulopathy, phobic postural vertigo, vertebro-basilar TIAs, acoustic neuroma, congenital disorders, enlarged vestibular aquaduct (EVA)-like or genetic disorders (like DFNA9), cervicogenic dizziness), based on the complete clinical record.
- Previous ear surgery for MD (IT injection is not an exclusion criterion)
- Language difficulties
- Active otitis media (with or without effusion)
- Unable or unwilling to use DizzyQuest App

- Unable to undergo MRI (such as gadolinium allergy, claustrophobia, implanted non-MRI compatible device of material, BMI)
- Deafness of the contralateral ear

- Rationale inclusion and exclusion

We aim to perform surgery only on patients with an active Ménière's disease. Because both procedures involve surgery with its associated risks, we only want to perform surgery on participants who have had no benefit of conservative medical therapy and IT injection. Moreover, this will reflect the group of patients who will undergo this surgery in usual clinical practice if this trial leads to EDB being a standard procedure. Patients with prior non-ablative surgery like ESD are excluded in order to avoid any influence in the end result. Moreover, we aim to eliminate or at least minimalize all factors that might interfere with follow-up. Patients with contralateral deafness are excluded because it is not clear what the effect of EDB on hearing function is.

4.4 Sample size calculation

The sample size for this RCT was computed using software package PASS 11. The sample size calculation is based on the study performed by Saliba (Saliba 2015), in which complete control of vertigo was reached in 96,5% of the patients who underwent EDB. According to literature, endolymphatic sac decompression is effective is ± 70% of the patients (Brinson 2007, Convert 2006, Durland 2006).

We compare MD participants undergoing an operation with clip (EDB group: A group) independently with MD participants undergoing operation without clip (decompressiongroup: B group). Null hypothesis is that the percentage points difference between groups percentages is nil (pA=pB), with two-sided alternative hypothesis (pA <> pB) and with anticipated 25% percent difference (pA= 95% and pB = 70%). Power is at least 80%. The chance of a false positive finding for either of the analyses is controlled at the 5% level (family wise error rate). To obtain a power of at least 80-% for Fisher's Exact test, the required sample size is 32 in groups A and B (allocation ratio =1). Loss to follow-up will likely occur in a small percentage of cases (n=5 per arm). No selective loss to follow-up is anticipated and a missing-at-random assumption seems reasonable. Missing outcomes will therefore be imputed using multiple imputation in the main analysis. Two sensitivity analyses will be conducted as well, where missing outcomes will be treated as failures or success, respectively. In this case the sample size would be 37 in group A and B (allocation ratio = 1). The total number of participants would be 74.

4.5 Numbers needed to treat

The number needed to treat (NNT) is an epidemiological measure used in communicating the effectiveness of a health-care intervention. The NNT is the average number of patients who need to be treated to prevent one additional bad outcome (e.g. the number of patients that need to be treated for one of them to benefit compared with a control in a clinical trial). For this study: NNT A vs B: 100/(p1-p2) = 100/(95%-70%) = 100/25 = 4

5. TREATMENT OF SUBJECTS

Participants in both arms of this study will undergo surgery. Both surgical procedures are described below.

5.1 Investigational treatment

The first part of the surgical procedure is equal in the two groups. First, a canal wall-up mastoidectomy is performed: the mastoid tegmen, sigmoid sinus, and sinodural angle are identified, and the posterior bony external ear canal wall is thinned. The posterior semi-circular canal (PSCC) and the dura mater of the posterior fossa are identified. Using the prominence of the horizontal semi-circular canal, Donaldson's line is identified to approximate the position of the endolymphatic sac. The bone over the sac and the dura are thinned with diamond burrs. The sac is completely skeletonized. The infralabyrinthine dura is exposed because the main body of the sac and its lumen often lie within this area. The bone of the vestibular aqueduct operculum is dissected. The posterior fossa dura from the retrolabyrinthine bone medial to the sac around the endolymphatic duct is exposed in order to identify the duct in its superior and inferior part in continuity from the endolymphatic sac, and to create a place to insert the tips of the instrument to clip the duct. At this level, care must be taken not to traumatize the dura, which is often thin. An (open) clip is then placed around the endolymphatic duct. After this placement, but before randomisation, a CT-scan of the petrous bone is performed to assess if the clip is adequately placed around the endolymphatic duct. If the duct is identified correctly and the clip is adequately placed, randomisation takes place.

- Surgical procedure in EDB group

If the patient is allocated to the EDB-group, the clip is closed. The size and numbers of clips used will be determined intraoperatively (Weck Horizon, size 'micro' to 'wide, Teleflex). The titanium clips are applied by using a clip applier (Weck Horizon) and a McGee bending forceps (Aesculap).
Theoretically, it is possible that the endolymphatic duct cannot be identified. In that case, the endolymphatic sac is separated from the posterior canal to ensure an arrest of the flow of endolymphatic fluid between the inner ear structures and the endolymphatic sac. This is done by placing a barrier of autologous temporal fascia glued with Tisseel. This way, the same result as blockage with a clip is obtained.
In the case of tearing of the dura leading to liquor leakage, this will be treated with tisseel, fascie and novacol. The cortex is not restored. The skin is closed in a regular way.

- Surgical procedure in endolymphatic sac decompression (control) group

The same surgical procedure is carried out in the decompression group. After identification of the endolymphatic duct, a CT scan is performed to assess if the endolymphatic duct was identified correctly. After ensuring the structure is indeed the duct, it is decompressed. In this group too, the cortex is not restored.

5.2 Use of co-intervention

Every patient will be treated by an experienced and skilled vestibular therapist after surgical intervention. The vestibular therapist will perform a functional assessment (including DGI, DVAT-NI, Romberg, sharpened Romberg, One Leg Stance Test and Motion Sensitivity Test) preoperatively. Based on the results, an individual tailored program will be made by the physiotherapist and the intensity of vestibular rehabilitation will be determined.
The program addresses patient functional deficits like visual, balance, gait and habituation disorders. Five 30-minute-sessions of vestibular rehabilitation will be given over a 3 months period. Training consists of habituation exercises (based on the Motion Sensitivity Test, developed by Shepard & Telian 1990), gaze stabilization exercises (according to Herdman 2014), postural stabilization exercises (balance training according to the framework of Klatt et al (2015)) and gait training according to the framework of Klatt et al. (2015) and Clendaniel, Tucci (1997). Apart from the sessions with the vestibular therapist, the patient must perform habituation and gaze stabilization exercises 2-3 times a day. Moreover, postural stabilization exercise and gait training must be performed 2-3 times a week.
If a patient has an increased risk of anxiety problems (HADS > 7), a written explanation will be given containing psychoeducation and advice of behaviour change based on cognitive-behaviour theory and self-regulation theory (according to Yardley and Kirby 2006).
The full vestibular rehabilitation treatment protocol can be found in the appendix (Appendix 3).

5.3 Escape medication

Medical indications or international guidelines about intervention after surgery are lacking. The decision to start any co-intervention will be based on the participants' experience of vertigo attack frequency and patient-doctor preference (shared decision-making). Shared decision-making ensures wide applicability of study results and reflects daily medical practice. The protocol for frequency and methods of IT injections can be found in the appendix (Appendix 4).

The use of metoclopramide in an acute phase of vertigo is allowed. The following dosages will be prescribed:

- Metoclopramide oral or rectal with a maximum of 30 mg per 24 hours

and/or

- Betahistin with a maximum of 48 mg per 24 hours or cinnerazine with a maximum of 75 mg per 24 hours

and/or

- Ondansetron with a maximum of 16 mg per 24 hours

Moreover, intervention may be carried out after surgery if the patient continues to suffer from vertigo attacks. This may be intratympanic injections of corticosteroids or in exceptional cases, ablative surgery such as vestibular neurectomy and labyrinthectomy. This is offered when the bouts of vertigo are intolerably frequent.

6. INVESTIGATIONAL PRODUCT

Not applicable.

7. NON-INVESTIGATIONAL PRODUCT

Not applicable.

8. METHODS

8.1 Study parameters/endpoints

8.1.1 Main study parameter

The main study parameter is the number of patients free of vertigo attacks at 12 months after surgery. This parameter was chosen because vertigo is the most invalidating symptom of MD.

8.1.2 Secondary study parameters

- Cumulative incidence of vertigo bouts
- Hearing
    - As defined in the diagnostic criteria of the Bárány Society (Lopez-Escamez, 2016), the following parameters will be assessed based on a three-tone audiometry at 0.5, 1 and 2 kHz (LFI):
        - The three-tone average score in decibels (dB), labelled as hearing threshold (HT).
        - Word recognition (WR), based on the speech discrimination. A decrease of ≥ 10 dB or a change in word recognition score of ≥ 15% points is considered clinically significant. When the pure-tone average and the WR change in the opposite direction, the pure-tone average score will be decisive to conclude the overall nature of change in hearing.
    - All pure tone audiometry analyses will be executed by the participating centre itself.
- Use of escape medication
    - The frequency and use of escape medication in the acute phase of vertigo will be recorded. This includes intratympanic injections and antivertigo medication.
- Co-interventions
    - Co-intervention is defined as performed ablative surgery during the follow up period because of unbearable vertigo bouts.
- Complications of surgery
- Functional Level Scale (FLS)
    - To assess the effect of the vertigo attacks on daily life, the 6-point FLS is recommended by the AAO-HNS for clinical trials in MD (Committee on Hearing and Equilibrium guidelines for the diagnosis and evaluation of therapy in Ménière's disease, 1995). One value is chosen to score the level of disability. The score will be reported as a total score, as

well as categorized into improvement, unchanged or worsened. Categories will be based on the following definitions:
        - Improvement: one or more points decrease on the FLS score compared to the baseline FLS score
        - Unchanged: unchanged FLS score compared to the baseline FLS score
        - Worsened: one or more points increase on the FLS score compared to the baseline FLS score.
- Dizziness Handicap Index (DHI)
    - To evaluate the effect of vertigo in the patient's daily life the 25-item measure of the DHI is used which was developed by Jacobson and Newman (1990). This inventory is often used for follow-up of participants suffering from dizziness. Higher scores indicate more severe handicap. According to a review published in 2007 (Duracinsky 2007) it is a valid tool and could be regarded as a questionnaire of reference.
    - The outcome is categorized as following:
        - 0-16 points: no handicap
        - 16-34 points: mild handicap
        - 36-52 points: moderate handicap
        - 54+: severe handicap
    - Categories will be based on the following definitions:
        - Improvement: the DHI score reflects at least 18 point improvement compared to baseline (Jacobson 1990).
        - Unchanged: the change in DHI score no greater than a 17 points increase of decrease compared at baseline.
        - Worsened: the DHI scores reflects a decrease of more than 17 points compared to baseline
- Tinnitus Handicap Index (THI)
    - To quantify the effect of tinnitus on daily life, the THI is used. The scale is grouped into functional, emotional, and catastrophic subscales. According to literature, the THI is a reliable and valid tool for measuring the impact of tinnitus on the quality of life (Newman (1996)), Kleinstäuber (2014)). The Dutch version of the questionnaire that will be taken is validated (Vereeck L 2006) It uses the following grades of handicap due to tinnitus:
        - 0-16: Slight or no handicap (Grade 1)
        - 18-36: Mild handicap (Grade 2)
        - 38-56: Moderate handicap (Grade 3)

        - 58-76: Severe handicap (Grade 4)
        - 78-100: Catastrophic handicap (Grade 5)
    - Categories will be based on the following definitions:
        - Improvement: decrease in grade of THI at 6 and 12 compared to the baseline THI grade.
        - Unchanged: unchanged THI grade compared to the baseline THI grade.
        - Worsened: increase on the THI grade compared to the baseline THI grade.
- SF-36
    - The SF-36 encompasses eight health concepts: physical functioning, bodily pain, role limitations due to physical health problems, role limitations due to personal or emotional problems, emotional well-being, social functioning, energy/fatigue, and general health perceptions. It also includes an item that provides an indication of perceived change in health.
      Scores are calibrated so that 50 is the average score or norm (Ware, 1993).
- EQ-5D VAS
    - The EQ-5D VAS records the respondent's self-rated health on a vertical, visual analogue scale where the endpoints are labelled 'best imaginable health state' and 'worst imaginable health state'.
- CEA
    - A trial-based cost-effectiveness analysis (costs per prevented vertigo-attack, from a healthcare perspective) and cost-utility analysis (costs per QALY, from a societal perspective), and cost-calculator spreadsheet model to estimate budget-impact from different perspectives will be performed using QALYs as the patient outcome. The EQ-5D and VAS score at 0, 3, 6 and 12 months will be used to assess quality of life.
- iPCQ
    - The productivity cost questionnaire (iPCQ) developed by Institute for Medical Technology Assessment (iMTA) assesses the impact of disease on the ability of a person to perform work. It is a standardized instrument for measuring productivity losses to enhance the comparability and generalizability of the outcomes of economic evaluations.
- iMCQ
    - The medical cost questionnaire (MCQ) developed by Institute for Medical Technology Assessment (iMTA) is used to measure health-care utilization. It

includes questions related to frequently occurring contacts with health care providers.

- BIA
  - Budget impact will be evaluated from the perspective of society, BKZ (‘budgettair kader zorg’), health insurer and the different specialists. The analysis will take into account the current mix of treatments with the annual number of procedures obtain from Vectis data.
- Endolymphatic hydrops and perilymph signal intensity on MRI
  - Recently developed MR-imaging allows visualization of perilymph signal intensity, and visualisation and grading of endolymphatic hydrops in de inner ear (Van Steekelenburg 2020). Hydrops is associated with duration of MD and saccular hydrops is associated with sensorineural hearing loss (Attyé 2018). Perilymphatic signal intensity is a surrogate marker for impaired blood-labyrinth permeability. Signal intensity (without) hydrops is markedly increased in the acute phase of labyrinthitis, and is increased in patients with MD (Shi, 2018). Overall, evidence is accumulating that both hydrops and signal intensity of the perilymph are associated with disease burden and may reflect disease activity. Therefore, we hypothesize that EDB results in a decrease in hydrops and perilymph signal intensity.
  - These two parameters will be measured pre-operatively, as well as 3-months and 12-months post-operatively to assess if the hydrops diminishes after EDB and is clinically relevant.
  - The protocol for the scans can be found in the appendix (Appendix 5)
- Physiotherapeutical outcomes
  - Balance
    - Assessment of static standing balance will be done by performing a Romberg, a sharpened Romberg and One Leg Stance Test. These tests are often used in the vestibular population (Gotshall 2011, Herdman 2014). Balance is a marker of vestibular function and it will be used as a guide in the vestibular rehabilitation. The results of the balance test will be compared with normative values (Gill-Body et al 2000, El Kashlan et al 1998)
    - The aim is to optimize balance ((sharpened) Romberg, one leg stance test), in which we strive to meet norm value or reaching a plateau for 6 weeks, within 3 months.
  - Gait

  - Gait will be assessed using the dynamic gait index (DGI), an instrument to evaluate an individual's ability to modify balance during walking. This tool is a marker of vestibular function, as an intact VOR is required to perform the tests.
  - The DGI contains 8 items which are all scored on a 4-point scale: 3 points for optimal score (no gait dysfunction), 2 points for minimal impairment, 1 point of moderate impairment and 0 points if there is a severe impairment in gait.
  - Improvement in gait is defined as 4.0 points (Marchetti 2014) improvement on the total of 24 points possible, compared to baseline.
- Dynamic Visual Acuity
  - Dynamic Visual Acuity will be measured using DVAT-NI. DVAT-NI measures the gaze stability by assessing the vestibulo-ocular reflex (VOR) function in response to examiner generated rotational head movement stimuli (2 Hz) during reading lines of the ETDR chart (SLOAN). The DVAT-NI is used to screen and characterize the severity of gaze instability.
  - Patient with a score of two or more line difference between static and dynamic acuity is defined as having an abnormal DVA (Dannenbaum 2009). The DVAT-NI is a clinical used test and used as a guide to treatment (Dannenbaum 2005, Dannebaum 2009, Herdman 2014) and to treatment efficacy.

8.2 Randomisation, blinding and treatment allocation

8.2.1 Process of randomisation

Participants will be allocated in the EDB group or endolymphatic sac decompression group using an automated telephone randomised service provided by Castor. Participants will be stratified according to gender and duration of MD (recent-onset versus mature MD participants). A "recent-onset MD participant" is defined as having their first MD vertigo attack in the last two years prior to inclusion. "Mature MD participants" have had their first MD vertigo attack more than two years prior to inclusion. By stratification for the duration of the disease, the effect of the natural course of disease on the outcome is reduced.

The two ear surgeons will be present up to wherein the sac is completely skeletonized. Then one of the surgeons will leave the OR. The randomisation for clip or decompression

operation will be performed using the automated telephone randomised service. The surgeon who leaves the OR will take care of the follow up and does not know whether the clip has been placed or not.

8.2.2 Blinding of randomisation and follow up

The ENT surgeon responsible for follow up will be blinded as he/she has left the OR when randomisation and possible clipping took place. The ENT surgeon will be blinded until deblinding at 1-year follow up of the last patient.
The coordinating investigator will take care of the follow up of the patients in the HagaHospital and will be blinded for these cases.

The patient will be blinded until the last patient is followed up for 1 year.

8.2.3 Emergency unblinding

Emergency unblinding may occur in the following situations:
- In case of a medical emergency where knowledge of the blinded treatment is necessary
- For the treatment of (serious) adverse event
- If requested by the Safety Committee.

The investigator must document the action taken and promptly notify the sponsor. Code breaks should only occur in exceptional circumstances as mentioned above and if it is absolutely essential for further management of the patient.

8.3 Study procedures

Throughout the trial, all data will be collected in Castor Electronic Data Capture (EDC). The handling of personal data will be in compliance with the Dutch General Data Protection Regulation (in Dutch: Algemene Verordening Gegevensbescherming (AVG)).

8.3.1 Preoperative procedures (T-1)

All PCs will be visited by the coordinating investigator (A.S.) for explanation about informing patients. A handout with information will be issued. This makes sure collection of data will be executed in a uniform and reproducible manner. Moreover, all principal investigators at each study site will be instructed which adverse events can be expected, how to inform the participants about the adverse events and how to process serious adverse events in both the eCRF and toetsingonline.nl (this is in line with the in the Ethical Committees guidelines in the Netherlands). All vertigo patients in the participating centres are registered, the fraction of Ménière’s patients is noted, the fraction of Ménière’s patients approached for participation is registered. This data is needed for generalisability.

Lastly, the data management system (Castor EDC) will be demonstrated and main investigators will fill out a questionnaire regarding their expectations of the trial.

PCs will assess patients for eligibility for the study using the inclusion and exclusion criteria. Once a patient has agreed to participate and has signed informed consent, a new record will be created in Castor by the coordinating researcher. Baseline characteristics, defined as the last valid values prior to study start or protocol initiation procedure, will be registered. Baseline characteristics that are entered into Castor:

Demographic data	Age	Years
	Gender	M/F
	Length	Cm
	Weight	Kg
	BMI	kg/m^2 (calculated)
Ménière's disease	First attack	dd-mm-yyyy
	Duration of MD	Days (calculated)
	Most recent attack	dd-mm-yyyy
	Mean number of attacks per month in the last 6 months	Number
	Previous treatments	None<br>Betahistine<br>Intratympanic injection
	Number of sessions IT injections	Number
	Number of IT injections	Number
	Substrate of IT injections	Dexamethason<br>Kenacort<br>Solumedrol
Comorbidities	All	(Free text)
In-/exclusion criteria	Meets in- and exclusion criteria	Yes/no
Attacks	Number of attacks in the past 6 months	Number
	Number of attacks in the past 2 months	Number
Informed consent	Informed consent obtained?	Yes/no
	Date of informed consent	dd-mm-yyy
	Version of IC-form	Free text

Physical examination	vHIT	Gain left/right
	Weber	No lateralization<br>Lateralizes to left<br>Lateralizes to right
	Rinne right	Positive<br>Negative
	Rinne left	Positive<br>Negative
	Dix Hallpike	Positive<br>Negative
	If positive: side:	Left<br>Right
	Diadochokinesis	Normal<br>Abnormal
	If abnormal: side	Left<br>Right
	Eye pursuit movements	Smooth<br>Abnormal
	If abnormal: direct	Left<br>Right<br>Up<br>Down

An MRI- and CT-scan will be performed and the patient is referred to a vestibular therapist. A PTA, vHIT and calorigram will be performed. Moreover, from the moment of inclusion, the patients starts reporting in de DizzyQuest App. This is a daily questionnaire of 31 questions addressing general functioning. In case of an attack, a short questionnaire is to be filled out. The inclusion and exclusion criteria will be double-checked by the coordinating investigator and if the patient does meet the criteria, the first survey package is sent through email. This survey pack consists of the HADS, DHI, THI, SF-36, EQ-5D VAS, NPQ, VADL[1], VAP[1] and a questionnaire about expectations of the treatment.
All the pre-operative data is entered in Castor.

[1] The VAP and VADL are newly developed questionnaires to assess the effect of dizziness and/or balance problems on patients' ability to do things. They are not an outcome measure in this study, but we aim to anticipate on the possibility that the Bárány Society takes the outcomes of questionnaires into account in future studies. These questionnaires are taken for this trial as well, to enable comparison of the results of this study in the future that only use the VAP and the VADL.

In the following weeks, the patient visits the vestibular therapist, who takes the medical history and performs the first measurements. The patient is seen by the anaesthetist for regular pre-operative screening.
The MRI, PTA, vHIT, calorigram and vestibular therapy are regular medical treatment. The CT-scan, reporting the DizzyQuest App and questionnaires are extra for this study. No diagnostic procedures will be postponed due to participation in this trial.

8.3.2 Surgical procedure (T=0)

Surgery takes place either at the site of inclusion, or in the HagaHospital. This depends on preference of the local researcher, and availability of equipment at the site. If surgery is performed at the HagaHospital, the local researcher (who has included and will follow the patient) is welcome to join the surgery. An experienced ENT-surgeon who has performed at least 20 EDB procedures (H.B.) will always attend surgery to ensure there are not intraoperative differences that may induce differences in outcome. Moreover, this facilitates blinding of the follow-up surgeon.
The surgical procedure is described in chapter 5 ‘Treatment of participants’, 5.1 Investigational product, subheading ‘surgical procedure in EDB group’. The randomisation for a clip or decompression operation will be performed using the automated telephone randomised service from Castor. The outcome of randomisation and placing of clip (only EDB) will be documented in Castor. For the purpose of ‘implantenregistratie’, all used clips and their LOT-number must be documented. Normally, this registration takes place in the electronic patient files, but for blinding purposes, this will now be documented in a paper notebook. After completion of the trial and deblinding, this information will be added to the electronic patient file.

An interim analysis of the data for the first 21 participants 6 weeks after surgery will be performed. The DSMB will assess the results and discuss the outcome, and give advice whether or not to continue the study.

8.3.3 Postoperative procedures

- One week after surgery (T+1)

One week after surgery, the patient will visit the ENT-surgeon for regular post-operative care. A vHIT will be performed. Moreover, the patient will visit the vestibular therapist whom will commence treatment. A balance test, dynamic visual acuity and gait analysis will be performed. Exercises that should be performed at home by the patient are explained. In the

following weeks, the patients carries these exercises out at home, and visit the vestibular therapist three times with two weeks in between the visits.
As surgery is currently not a normal medical treatment, this visit and these measurements are all extra for this study.

- Three months after surgery (T+2)

At three months after surgery, a follow up visit is planned at the ENT-surgeon. A vHIT is performed. The patients visits the vestibular therapist who performed measurements who finishes treatment and performs measurements. An MRI-scan and PTA are performed. The patients receives a survey package through email, consisting of the DHI, THI, FLS, EQ-5D VAS, SF-36, iMCQ and iPCQ.
This visit and these measurements/tests are all extra for this study.

- Six months after surgery (T+3)

At six months after surgery, a follow up visit is planned at the ENT-surgeon. A vHIT is performed. The vestibular therapist performs measurements. A PTA is performed. The patients receives a survey package through email, consisting of the DHI, THI, FLS, EQ-5D VAS, SF-36, iMCQ and iPCQ.
This visit and these measurements/tests are all extra for this study.

- Twelve months after surgery (T+4)

At twelve months after surgery, the last follow up visit for this trial is planned. A vHIT is performed, as well as a PTA, an MRI-scan and calorigram. The vestibular therapist performs measurements and the patients receives a survey package through email consisting of the DHI, THI, FLS, EQ-5D VAS, SF-36, iMCQ, iPCQ, VADL and VAP. If the patient was in de EDB-group, a CT-scan will be performed to assess if the clip is in place in the patients still suffers attacks. If results are in favour of EDB, and patients still suffer attacks, the patients in the decompression group will be offered EDB surgery.
This visit and these measurements/tests are all extra for this study.

8.3.4 Flow chart

A simplified flowchart can be found here. For the more specified flow chart, see Appendix 2.

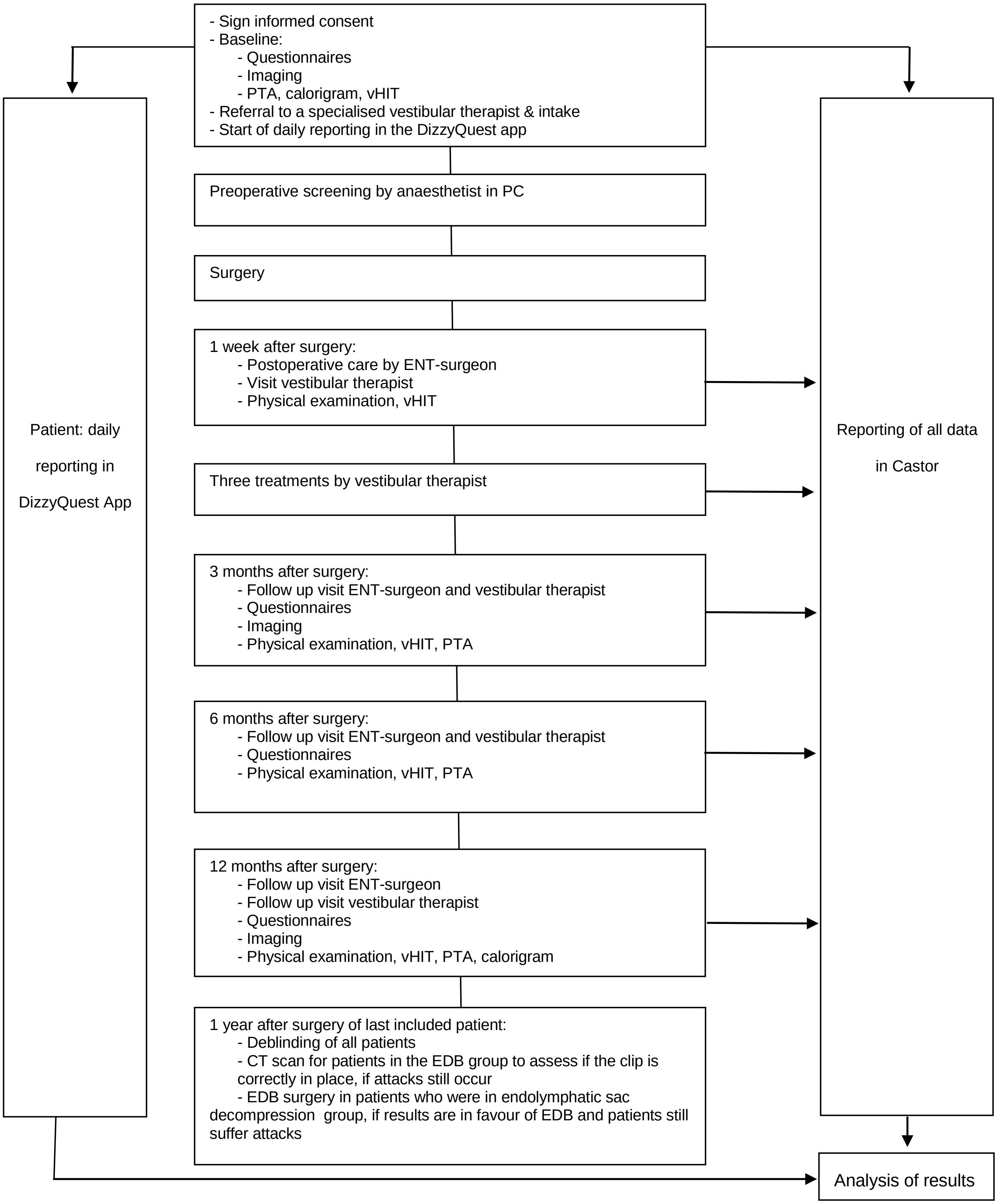

8.4 Withdrawal of individual subjects

Participants can leave the study at any time for any reason if they wish to do so without consequences. The investigator can decide to withdraw a patient from the study for urgent medical reasons.

8.5 Replacement of individual subjects after withdrawal

The number of participants (as mentioned in chapter 3) takes into account that at least 15% of the participants in both arms of the study will drop out. We therefore do not expect to replace participants.

8.6 Follow-up of subjects withdrawn from treatment

A participant may be excluded from the study prior to the expected protocol completion for safety reasons, failure of subject to adhere to protocol requirements, or subject consent withdrawal. Since early withdrawal could be related to the surgery, it is of great importance to analyse these final data for the integrity of the study. If a participant withdraws consent to continue follow up participation, attempts should be made to obtain permission to record at least primary outcome data. The participant will be contacted by phone and email, the next-of-kin will be contacted, a letter asking to contact us will be send or the patient’s general practitioner will be contacted.

If a participants withdraws its consent, he/she is allowed to do so without further explanation. If this is the case, participants should contact the ENT-surgeon who takes care of follow up. This ENT-surgeon then contacts the coordinating researcher. The data that were collected until the moment of consent withdrawal, will be used in the analyses.

8.7 Premature termination of the study

Termination of the study will be considered in the case of more than 2 patients with facial nerve damage and/or severe hearing loss, as a result of surgical damage of the facial canal or posterior canal during the operation.
Depending on the results of the interim analysis, the DSMB can advise to terminate the study.

9. SAFETY REPORTING

9.1 Temporary halt for reasons of subject safety

In accordance to section 10, subsection 4 of the WMO, the sponsor will suspend the study if there is sufficient ground to assume that continuation of the study will jeopardise patient health or safety. The sponsor will notify the accredited METC without undue delay of a temporary halt including the reason for such an action. The study will be suspended pending a further positive decision by the accredited METC. The main investigator (A.S.) will keep the participants informed.
Interim analysis will be performed after surgical treatment of 21 participants. In case of more than 5 adverse events (such as permanent hearing loss, extra hospitalisation after surgery), the METC will be notified. In a serious adverse event, the METC will be informed within the legal requirements (< 24 hour).

Substantial protocol changes will be discussed and presented for approval by METC-LDD.

9.2 AEs, SAEs and SUSARs

9.2.1 Adverse events (AEs)

Adverse events are defined as any undesirable experience occurring to a patient during the study, whether or not considered related to the performed (EDB/endolymphatic sac decompression) surgery. All adverse events reported spontaneously by the patient or observed by the investigator or her staff will be recorded.

As with every surgical procedure, the operation that patients undergo comes with risks. Data in a systematic review and meta-analysis regarding perioperative and anaesthetic related mortality suggest a mortality of 34 per million (Bainbridge, 2012).

The specific risks of this surgical procedure are:
- Injury to the facial nerve: according to Saliba's protocol (2015) this occurs in less than 1% of the patients.
- Injury to posterior semi-circular canal with loss of vestibular function
- Injury to the sigmoid sinus
- Hearing loss: in less than 4% of the patients, permanent hearing loss of the operated ear was reported (Saliba, protocol 2015).
- Leakage of cerebrospinal fluid (CSF). Minor leakage occurred in 10-15 %. This was solved intraoperatively by filling with muscle autograft temporalis fascia with biological glue. There

were no postoperative complications due to the leakage (Saliba's protocol 2015; Asmar 2016). It is important to note that the surgery procedure was optimized by Blom. We expect that this results in a lower rate of CSF leaks. Blom follows the dura of the posterior cranial fossa, and has good vision at the horizontal semicircular canal after visualising Donaldson's line.

- Meningitis
- According to the study performed by Saliba, 11 out of 57 patients experienced a post-operative benign paroxysmal positional vertigo (BPPV) (Saliba, 2015). In the pilot group of 66 patients operated by Blom, BPPV was not reported. The difference in BPPV incidence may be due to the enhanced technique; Saliba works towards the posterior canal whilst Blom follows the dura of the posterior fossa and usual does not blue line the posterior canal.

Adverse effects due to natural course of MD are:

- worsening of hearing loss
- worsening of vertigo
- occurrence of bilateral MD
- worsening of tinnitus

9.2.2 Serious adverse events (SAEs)

A serious adverse event is any untoward medical occurrence or effect that

- results in death;
- is life threatening (at the time of the event);
- requires hospitalisation or prolongation of existing inpatients' hospitalisation;
- results in persistent or significant disability or incapacity;
- any other important medical event that did not result in any of the outcomes listed above due to medical or surgical intervention but could have been based upon appropriate judgement by the investigator.

An elective hospital admission will not be considered as a serious adverse event.

The investigator will report all SAEs to the sponsor without undue delay after obtaining knowledge of the events.

The sponsor will report the SAEs through the web portal *ToetsingOnline* to the accredited METC that approved the protocol, within 7 days of first knowledge for SAEs that result in death or are life threatening followed by a period of maximum of 8 days to complete the initial

preliminary report. All other SAEs will be reported within a period of maximum 15 days after the sponsor has first knowledge of the serious adverse events.

9.2.3 Suspected unexpected serious adverse reactions (SUSARs)

NA.

9.3 Annual safety report

NA.

9.4 Follow-up of adverse events

All AEs will be followed until they have abated, or until a stable situation has been reached. Depending on the event, follow up may require additional tests or medical procedures as indicated, and/or referral to the general physician or a medical specialist.
SAEs need to be reported until the end of study within the Netherlands.

9.5 Data Safety Monitoring Board (DSMB)

A Data Safety Monitoring Board (DSMB) will be established. It will be independent from the investigators and the sponsor.
Members of the DSMB:
Dr. A. van Hylckama Vlieg (epidemiologist, LUMC)
Dr. Ir. N. van Geloven (statistician, LUMC)
Dr. M. Hol (experienced clinician in the field, Radboud UMC/Groningen UMC)

The members are not in any other way involved in the trial and have therefore no conflict of interest with the sponsor of the study.
Members will review the interim analyses for the first 21 participants 6 weeks after surgery, focussing on safety of the surgical procedures.
Termination of the trial will be considered if there are more than 2 patients with facial nerve damage and/or severe hearing loss as a result of surgical damage of the facial canal or posterior canal during the operation. The trial will be stopped in case of futility, when it seems to be unlikely that there is a clinical significant effect based on the results of the interim analysis results.
The advice(s) of the DSMB will be sent to the principal investigator of the study. Should the sponsor decide not to fully implement the advice of the committee, the sponsor will send the

advice to the reviewing METC, including a note to substantiate why (part of) the advice of the committee will not be followed.

During the time when interim analysis takes place, no new participants will be included in this study.

An extensive charter for the DSMB can be found in chapter K5 ‘Overige documenten’ of this dossier.

10. STATISTICAL ANALYSIS

Statistical Data analysis of the study data will be performed using SPSS (version 17.0). All analyses will be performed through Intention to treat.

10.1 Primary study parameter

The primary outcome is defined as being attack free at 12 months follow-up. The required significance level of findings (α) will be equal to or lower than 5%. Any statistical tests will be two-sided. P-values will be presented. Where confidence limits can be estimated, the confidence level will be 95%. All analyses will be performed by intention to treat.
A chi-square test (or Fisher's exact test) will be performed on the primary outcome variable data (number of patients free of vertigo attacks at 12 months post-operatively, in EDB vs endolymphatic sac decompression group).

10.1.1 Imputation of App-recorded data

The daily questionnaire taken via the DizzyQuest app is likely to contain missing data. All other missing data will be labelled 'NAmissing' in SPSS. Multiple imputation will be used to create complete data sets. Depending on the missing data pattern different strategies will be followed.
Imputation variables are base line measurements, time-course measurements and daily App-data.

- Missingness ≤ 10%

If little data is missing, the data will be treated as one data-row per patient (wide format). In this case, information for imputing missing values will be taken from the exact same time points of other patients. This strategy can account for possible time trends.

- Missingness > 10%, ≤ 50%

If more data is missing, the data of each patient will be split into one row per day (long format), when the previous 10 days will be included in each row. After imputation, the previous days will be removed to avoid duplication of data. This strategy includes information on missing data across time periods and can lead to wrong imputations for time trends longer than 10 days.

- Missingness > 50%

The same strategy as for missingness between 10% and 50% is followed except for including a smaller time window. 10 previous days will be used. If all primary outcome data are not available (missing), the participant will be excluded from the analyses. The a priori sample size estimation has been corrected for this.

The above cut-offs and time ranges reflect expectations. Actual values will be selected based on the variability of imputed values.

10.1.2 Computation of outcome

The outcome will be determined from the imputed App-data. It is expected that attacks are reported reliably and missing data can be reliably imputed as being attack free. In principle, a patient can be sometimes imputed as having an attack on otherwise as being attack-free. To account for these potential cases, a cut-off of 10% for the attack probability (as the imputed frequency for having an attack) will be used to determine presence/absence of attacks.

10.2 Secondary study parameters

The Patient Reported Outcome Measures (PROMs) used in this study are assumed to be continuous numerical and will be tested checked for near-Gaussian distribution normality before analysis. Results will be described as means (with 95%CI) in case of near-Gaussian distribution or, otherwise medians (with IQR) will be presented at each time point. Categorical outcomes will be presented in numbers of participants with accompanying percentages of group total.

10.2.1 Missing data

For the missing data in the various questionnaires, we use the standard rules concerning missing data.

10.2.2 Computation of outcome

All secondary outcomes will be analysed using a linear mixed model (EDB vs decompression group) at the different time measurement point.

10.3 Other study parameters

NA

10.4 Interim analysis

An interim analysis of the data for the first 21 participants will be performed after 6 weeks follow up of the 21st surgery. The DSMB will discuss the safety of the participants, and advice whether or not to continue the study.

11. ETHICAL CONSIDERATIONS

11.1 Regulation statement

The study will be conducted according to the principles of the Declaration of Helsinki (October 2013) and in accordance with the Medical Research Involving Human Subjects Act (WMO, 26 February 1998) and the International Conference on Harmonization Good Clinical Practice (ICH GCP, November 2016). The study will be conducted in compliance with the protocol.

11.2 Recruitment and consent

Patients with MD meeting the inclusion criteria will be asked to participate in the study by their ENT specialist in the PC. Consent to enter the study must be sought from each participant only after a full explanation by the ENT specialists of the PC has been given, an information leaflet offered, and time allowed for consideration (one month at maximum). Patients will especially be informed that two slightly different surgical procedures will be performed.

Patients are allowed to contact the independent expert for any questions. Signed participant consent should be obtained. The ENT specialists of the PC also signs the consent form. The right of the patient to refuse to participate without giving reasons must be respected at all times throughout the trial.

11.3 Objection by minors or incapacitated subject

Not applicable.

11.4 Benefits and risks assessment, group relatedness

Both the EDB and decompression surgery are not without its risks.

A mastoidectomy and identification of the endolymphatic duct could lead to meningitis, hearing loss, facial nerve palsy, labyrinth function loss. Meningitis and facial nerve palsy have not been reported (nor literature nor in the pilot groups operated in Antwerp by Blom). Mild hearing loss has been seen in 2 patients in our pilot. Three patients of the pilot group treated with EDB suffered drop attacks, but recovered within 8 weeks. Moreover, in three patients operated in Antwerp, postsurgical CSF leakage occurred, but successful surgical reintervention was performed the next day.

Benefits for the participants in this study are that 50% of the participants will undergo EDB surgery of which we believe (based on the previously discussed results of EDB) that it will effectively treat their vertigo attacks. Of the participants in the decompression group, an

estimated 70% will benefit from the surgery. Therefore, only 30% of the decompression group (n = 13, 15% of the total study population) will not directly benefit from participation in the study.
Furthermore, if at the end of the study these participants would still be eligible for EDB surgery and results are in favour of the EDB procedure, EDB surgery can be performed in less time with less trauma and risk, since the access to the ED through the petrous bone has already been established during the decompression operation.

Depending on the group the participant is in, 2 or 3 CT scan will be performed.
In the EDB group, one CT scan will be performed preoperatively to assess the course of the endolymphatic duct, which is helpful preoperatively in finding the duct. After placement of the clip, a CT scan is performed to assess the clip position. After deblinding, a third CT scan can be performed to assess clip position, but only if the participant still has vertigo attacks.
In the endolymphatic sac decompression group, the same preoperative CT scan will be performed. The same peroperative CT-scan is performed to asses if structures were correctly identified.
Each performed CT scan leads to exposure to ionizing radiation: approximately 2 mSv per scan. For reference: the background radiation in the Netherlands is approximately 2.5 mSv per year.

11.5 Compensation for injury

The sponsor/investigator has a liability insurance that is in accordance with article 7, subsection 6 of the WMO. This is through the insurance company of the HagaHospital (Centramed). The sponsor also has an insurance, which is in accordance with the legal requirements in the Netherlands (Article 7 WMO and the Measure regarding Compulsory Insurance for Clinical Research in Humans of 23th June 2003). This insurance provides coverage for damage to research participants through injury or death caused by the study. The insurance applies to the damage that becomes apparent during the study or within 4 years after the end of the study.

11.6 Incentives

None.

11.7 Other ethical considerations

11.7.1 Support of other relevant communities

Support of relevant patient community, the Dutch ENT Society (KNO-vereniging) and the vertigo centres is obtained. The relevant patient community has endorsed the protocol and the patient information. Their written support can be found in this dossier in Chapter K, ‘Overige documenten’.

12. ADMINISTRATIVE ASPECTS, MONITORING AND PUBLICATION

12.1 Handling and storage of data and documents

- Castor EDC

Data storage will be done in Castor, which provides electronic data capturing. Castor is secured according to the most recent standards in order to protect data and is certified for ISO 27001 (Standards for Information Security Assurance). The handling of personal data will be in compliance with the EU General Data Protection Regulation and the Dutch Act on Implementation of the General Data Protection Regulation (in Dutch: Uitvoeringswet AVG, UAVG).
Building of the database in Castor will be done by the coordinating investigator initiating centre (HagaZiekenhuis) under supervision of a trained data manager. The coordinating investigator will have access to all patient records of the participating centres. She will be the only individual to enter data into Castor EDC.
In case of unexpected, long-term absence of the coordinating investigator, the data entry will be performed by the local investigators. Access to the database and rights for data entry of only their own centre will be provided before the start of the trial.

Per centre, a subject identification list will be safeguarded by the main investigator of the centre. The local study team can access this file. Coding of participants will not include patient initials or birthdate.

The coordinating investigator will have (secured, remote) access to the patient files of each participating centre. All data will be extracted from the patient records and recorded in Castor EDC.

The MRI scans will be sent on a CD-ROM by registered mail (as is usual clinical practice) to the HagaHospital for double assessment. After scanning in the images, the CD-ROM will be destroyed. The second radiologist will assess the images in the HagaHospital and note the findings in the records of the HagaHospital. From there, the coordinating investigator will record the data in Castor.

The only persons having access to the source data will be the local investigator, the local study team, the coordinating investigator, the monitor and possibly the inspector.

The only person having access to Castor EDC is the coordinating investigator and possibly researches from the local study team. All persons with access to Castor EDC will have

access restricted to only the necessary parts of Castor, i.e. only to the data of patients from their own centre. Moreover, there will be one other person with access to the data, for the case of unexpected events (such as unavailability of the coordinating investigator). Access to the data will be logged, and apart from a short training, this person will not be looking into the data. This ‘back up’ person will be J.P. Koopman, ENT-surgeon in the HagaHospital, who is not in any other way involved in the trial.
All data will be stored in Castor for 15 years after the last subject has had the last study visit. The servers are located in Amsterdam.
Members of the research team will be informed in case record retention is prolonged or when records are no longer needed.

- DizzyQuest app

There will be a ‘verwerkingsovereenkomst’ between the HagaHospital and the builder of the DizzyQuest App (Psymate) in which all handling of data will be recorded. Data in the DizzyQuest app will be stored on the smartphone of the user until a stable internet connection is established. Data saved on the smartphone is encrypted and not accessible for third parties. When the smartphone connects to the internet, data will be transferred to the servers of Smart eHealth, located in Belgium. After transfer of the data, all data will be deleted automatically from the smartphone.

The coordinating investigator will monitor appliance to the DizzyQuest app. Every night, the servers generate an automatic email that is sent to the coordinating investigator, containing general information on status of participants. Therefore, if participants miss questionnaires, this will detected and the participant will be contacted through phone or email.

The only person having access to all the data from the app is the coordinating investigator. Here too J.P. Koopman will have access for the case of unexpected events. Access to the data is logged. The administrator of the database will have access to the data for quality and security control. However, email-addresses of the patient cannot be seen by the administrator. All access provisions are specified in the ‘verwerkingsovereenkomst’.

After termination of the one-year follow up period, participants can delete the DizzyQuest app from their smartphone. When the app is deleted, all data are deleted from the phone as well. All data will be safely transferred to the servers of the HagaHospital after termination of the trial. Here, data will be saved for 15 years. This will be recorded in the processing agreement between PsyMate and the HagaHospital.

12.2 Monitoring and Quality Assurance

Monitoring of the conduct of the study will be performed according to the monitoring plan of the Haga Science Department, which can be found in the appendix (Appendix 6). In the HagaHospital, this will be performed by the HagaAcademy. In the other centres, monitoring will be performed by the monitor of the Haga Hospital, in compliance with the monitoring plan.
The monitor makes a monitoring report for the principal investigator after each visit. The original monitoring reports are stored at the Castor Management System.

- Monitoring of DizzyQuest App

Monitoring of compliance to the DizzyQuest will be performed by the coordinating investigator (AS). She is responsible for transportation of the data into Castor.

- Monitoring of questionnaires sent through Castor

The coordinating investigator will monitor if patients fill in the questionnaires in Castor. If necessary, email reminders can be sent to the patient through Castor. If necessary, patients will be called.

12.3 Amendments

Amendments are changes made to the research after a favourable opinion by the accredited METC has been given. All amendments will be notified to the METC that gave a favourable opinion.

12.4 Annual progress report

The investigator will submit a summary of the progress of the trial to the accredited METC once a year. Information will be provided on the date of inclusion of the first subject, numbers of subjects included and numbers of subjects that have completed the trial, serious adverse events/ serious adverse reactions, other problems, and amendments.

12.5 Temporary halt and (prematurely) end of study report

The investigator/sponsor will notify the accredited METC of the end of the study within a period of 8 weeks. The end of the study is defined as the last patient's last visit at one-year follow up.

The sponsor will notify the METC immediately of a temporary halt of the study, including the reason of such an action.

In case the study is ended prematurely, the sponsor will notify the accredited METC within 15 days, including the reasons for the premature termination.

Within one year after the end of the study, the investigator/sponsor will submit a final study report with the results of the study, including any publications/abstracts of the study, to the accredited METC.

12.6 Public disclosure and publication policy

Results of this study will be published in international peer-reviewed scientific journals and will be presented on (inter)national scientific conferences and meetings. This will be in accordance to the CCMO statement on publication policy.’

Individual centres included in this multicentre trial are not allowed to report or publish data from this centre alone. Transfer of ownership of the data will be reported to the appropriate authority/authorities, as required by the applicable regulatory requirement(s). All publications and presentations are to protect the research integrity of the participants and objectives of the study. No data will be presented of released that may break the masking of the study trial. The timing of presentation and/or publications of the primary and/or secondary outcomes will be secured by the supervising researchers and will be communicated first with all centres involved.

12.6.1 Data analysis and release of results

The data from the trial will be centrally analysed from Participating Centres by the trial statistician.
We plan to use non-parametric tests in the analysis of the data. Non-parametric methods are more robust as they make fewer assumptions (distribution of the data). As a downside non-parametric are less efficient, i.e. they need a higher sample size to achieve the same power. As a compensating factor we use the relative efficiency of the Mann-Whitney U-test as compared to the t-test which has been estimated as 95% (Conover 1999, Practical Nonparametric Statistics).

In order to estimate a realistic treatment effect that avoids bias due to treatment-arm crossover and selective drop-out, an intention-to-treat analysis will be conducted as the primary analysis.

12.6.2 Review process

Each article or abstract will be submitted according to the authors' guideline as predefined by the journal. Before submitting, the supervising researchers will recommend changes to the authors. A pre-submission English-language editing will be performed.

12.6.3 Close-out procedures

The EDB study is expected to end 1 year after the last patient has been operated. Regardless of the timing and circumstances of the end of the study, the close-out procedure will be executed in two stages:

- Interim period for analysis and documentation of study results. The time between completion of the data collections and the release of the study results will be minimalised. It is expected that it will take about 6 months after completion of follow up of the last patient for the results to be submitted to an appropriate journal.
- Zorginstituut Nederland will evaluate the results and decide if the intervention should be implemented in Dutch health care.

13. STRUCTURED RISK ANALYSIS

Not applicable.

14. REFERENCES

Adrion C, Fischer CS, Wagner J, et al. Efficacy and safety of betahistine treatment in patients with Ménière's disease: primary results of a long term, multicentre, double blind, randomised, placebo controlled, dose defi ning trial (BEMED trial). BMJ 2016; 352: h6816.

Alexander JH, Harris JP. Current epidemiology of Ménière's syndrome. Otolaryngol Clin N Am 2010;43:965-970.

Alghwiri AA, Whitney SL, Baker CE, Sparto PJ, Marchetti GF, Rogers JC, Furman JM. The development and validation of the vestibular activities and participation measure. Arch Phys Med Rehabil. 2012 Oct;93(10):1822-31.

Asmar MH, Saliba I, Endolymphatic Duct Blockage for Refractory Ménière's Disease: Assessment of Endolymphatic Sac Biopsy on Short-Term Surgical Outcomes, J Int Adv Otol 2016; 12(3): 310-5 • DOI: 10.5152/iao.2016.3069

Attyé A, Eliezer M, Medici M, et al. In vivo imaging of saccular hydrops in humans reflects sensorineural hearing loss rather than Ménière's disease symptoms. Eur Radiol 2018;28:2916–22.

Bainbridge D, Martin J, Arango M, Cheng D; Evidence-based Peri-operative Clinical Outcomes Research (EPiCOR) Group. Perioperative and anaesthetic-related mortality in developed and developing countries: a systematic review and meta-analysis. Lancet. 2012 Sep 22;380(9847):1075-81.

Baráth K et al. Detection and grading of endolymphatic hydrops in Ménière disease using MR Imaging. AJNR Am J Neuroradiol 2014;35:1387-1392.

Basura GJ, Adams ME, Monfared A, et al. Clinical Practice Guideline: Ménière's Disease. Otolaryngology--head and Neck Surgery : Official Journal of American Academy of Otolaryngology-head and Neck Surgery. 2020 Apr;162(2_suppl):S1-S55. DOI: 10.1177/0194599820909438.Bernaerts A, Vanspauwen R, Blaivie C, et al. The value of four stage vestibular hydrops grading and asymmetric perilymphatic enhancement in the diagnosis of Ménière ' s disease on MRI. Neuroradiology 2019;61:421–9.

Bouma et al. Duizeligheid, NHG-standaard Nederlands Huisartsen Genootschap, 2017.

Bretlau P, Thomsen J, Tos M, Johnsen NJ. Placebo effect in surgery for Ménière's disease: nine-year follow-up. American Journal of Otology 1989;10(4):259–61.

Brinson GM, Chen DA, Arriaga MA. Endolymphatic mastoid shunt versus endolymphatic sac decompression for Ménière's disease. Otolaryngol Head Neck Surg. 2007;136(3):415-421. doi:10.1016/j.otohns.2006.08.031

Bojrab DI 2nd, LaRouere MJ, Bojrab DI, Babu SC, Sargent EW, Chan EY, Hong RS. Endolymphatic Sac Decompression With Intra-Sac Dexamethasone Injection in Menière's

Disease. Otol Neurotol. 2018 Jun;39(5):616-621. doi: 10.1097/MAO.0000000000001810. PMID: 29738389.

Convert C, Franco-Vidal V, Bebear JP, Darrouzet V. Outcome-based assessment of endolymphatic sac decompression for Ménière's disease using the Ménière's disease outcome questionnaire: a review of 90 patients. Otol Neurotol. 2006 Aug;27(5):687-96. doi: 10.1097/01.mao.0000227661.52760.f1. PMID: 16868517.

Chung JW, Fayad J, Linthicum F. Histopathology after endolymphatic sac surgery for Ménière's syndrome. Otol Neurotol. 2011;32:660-664.

Clendaniel RA, Tucci DL.(1997) Vestibular rehabilitation strategies in Ménière's disease. Otolaryngologic clinics of North America, vol 30, nr 6, 1145-1158

Committee on Hearing and Equilibrium guidelines for the diagnosis and evaluation of therapy in Ménière's disease, American Academy of Otolaryngology-Head and Neck Foundation, Inc. Otolaryngol Head Neck Surg. 1995 Sep;113(3):181-5.

Conover 1999, Practical Nonparametric Statistics, ISBN: 978-0-471-16068-7

Dannenbaum E1, Paquet N, Hakim-Zadeh R, Feldman AG. Optimal parameters for the clinical test of dynamic visual acuity in patients with a unilateral vestibular deficit. J Otolaryngol. 2005 Feb;34(1):13-9.

Dannenbaum, E. Paquet, N. et al (2009). Clinical evaluation of dynamic visual acuity in subjects with unilateral vestibular hypofunction. Otol Neurotol 30(3): 368-372.

Duracinsky M., Mosnier I., Bouccara D., Sterkers O., Chassany O., Attal N.,Toupet M. (2007). Literature review of questionnaires assessing vertigo and dizziness, and their impact on patients' quality of life. Value in Health. (Vol 10 pp. 273–284).

Durland WF Jr, Pyle GM, Connor NP. Endolymphatic sac decompression as a treatment for Ménière's disease. Laryngoscope. 2005;115(8):1454-1457. doi:10.1097/01.mlg.0000171017.41592.d0

El Kashlan, H.K., Shepard, N.T. et al (1998). "Evaluation of clinical measures of equilibrium". Laryngoscope 108(3): 311-319.

Van Esch BF et al. Age of onset of Ménière's disease in the Netherlands: data from a specialised dizziness clinic. J Laryngol Otol. 2016 Jul;130(7):624-7.

Van Esch BF et al. Two common second causes of dizziness in patients with Ménière disease. Otology and Neurotology 2016 Dec;37(10):1620-1624

Friis M, Thomsen AR, Poulsen SS, Qvortrup K. Experimental hyperactivity of the endolymphatic sac. Audiol Neurootol. 2013;18:125-133.

Gabra N, Asmar MH, Berbiche D, Saliba I. Endolymphatic duct blockage: quality of life assessment of a novel surgical technique for MD. Eur Arch Otorhinolaryngol. (2016)273:2965–2973 [Epub ahead of print] PubMed

Gill-Body, K.M., Beninato, M. et al (2000). "Relationship among balance impairments, functional performance, and disabilty in people with peripheral vestibular hypofunction." Physical Therapy 80 (8); 748-758.

Gottshall K. (2011). Vestibular rehabilitation after mild traumatic brain injury with vestibular pathology. NeuroRehabilitation 29(2): 167-171.

Harner SG, Driscoll CL, Facer GW, Beatty CW, McDonald TJ. Long-term follow-up of transtympanic gentamicin for Ménière's syndrome. Otol Neurotol. 2001 Mar;22(2):210-4.

Herdman SJ, Clendaniel RA. Vestibular Rehabilitation, 4 th edition, 2014.

Huang T, Lin C, Chang Y. Endolymphatic sac surgery for Ménière’s disease. Acta Otolaryngol Suppl. 1991;485:145-154.

Jacobson GP, & Newman CW (1990). The development of the Dizziness Handicap Inventory. Archives of Otolaryngology--Head & Neck Surgery. (Vol 116 pp. 424–427).

Kitahara T, Horii A, Imai T, Ohta Y, Morihana T, Inohara H, Sakagami M. Effects of endolymphatic sac decompression surgery on vertigo and hearing in patients with bilateral Ménière's disease. Otol Neurotol. 2014 Dec;35(10):1852-7. doi: 10.1097/MAO.0000000000000469. PMID: 24979126.

Klatt BN, Carender WJ, Lin CC, Alsubaie SF, Kinnaird CR, Sienko KH, Whitney SL. (2015) A Conceptual Framework for the Progression of Balance Exercises in Persons with Balance and Vestibular Disorders. Phys Med Rehabil Int; 2(4).

Kleinstäuber M., Frank I., & Weise C. (2014). A confirmatory factor analytic validation of the Tinnitus Handicap Inventory. Journal of Psychosomatic Research.

Kotimaki J, Sorri M, Aantaa E, Nuutinen J: Prevalence of Ménière disease in Finland. Laryngoscope , 1999;109:748–753

Linthicum FH, Santos F. Endolymphatic sac amputation without hydrops. Otol Neurotol. 2011;32:e12-e13.

Lopez-Escamez JA, Carey J, Chung WH, et al. Diagnostic criteria for Ménière's disease. Consensus document of the Bárány Society, the Japan Society for Equilibrium Research, the European Academy of Otology and Neurotology (EAONO), the American Academy of Otolaryngology-Head and Neck Surgery (AAO-HNS) and the Korean Balance Society. Acta Otorrinolaringol Esp. 2016;67(1):1-7. doi:10.1016

Marchetti GF, Chia-Cheng L, Alghadir A, Whitney SL.(2014). Responsiveness and minimal detectable change of the dynamic gait index and functional gait index in persons with balance and vestibular disorders. J Neurol Phys Ther. 2014 Apr;38(2):119-24.

Mateijsen, D.J.M. (2001). Definition Ménière Groningen: A rational approach to Ménière’s disease (Proefschrift). Groningen: Rijksuniversiteit Groningengoscope, 94, 1098-1102.

McRackan TR1, Best J, Pearce EC, Bennett ML, Dietrich M, Wanna GB, Haynes DS, Labadie RF. Intratympanic dexamethasone as a symptomatic treatment for Ménière's disease. Otol Neurotol. 2014 Oct;35(9):1638-40.

Merchant `SN, Adams JC, Pathophysiology of Ménière's syndrome: are symptoms caused by endolymphatic hydrops? Otol Neurotol. 2005; 26(1):74-81

Naganawana et al. Visualization of endolymphatic hydrops with MR imaging in patients with Ménière’s disease and related pathologies: current status of its methods and clinical significance. Jpn J Radiol 2014;32;191-204

Nakashima T, Naganawa S, et al. Visualization of endolymphatic hydrops in patients with Ménière's disease. Laryngoscope. 2007 Mar;117(3):415-20.

Nederlandse Vereniging voor Keel-Neus-Oorheelkunde en Heelkunde van het Hoofd-Halsgebied, Richtlijn ‘Duizeligheid bij ouderen’. 2015

Newman C. W., Jacobson G. P., & Spitzer J. B. (1996). Development of the Tinnitus Handicap Inventory. Archives of Otolaryngology--Head & Neck Surgery. (Vol 122 pp. 143–148).

Patel, Mitesh et al., Intratympanic methylprednisolone versus gentamicin in patients with unilateral Ménière's disease: a randomised, double-blind, comparative effectiveness trial. The Lancet , 2016, Volume 388 , Issue 10061 , 2753 – 2762

Perez-Garrigues H, Lopez-Escamez J, et al. Time course of episodes of definitive vertigo in Ménière's disease. Arch Otolaryngol Head Neck Surg. 2008 Nov;134(11):1149-54.

Portmann G. The old and new in Ménière’s disease - over 60 years in retrospect and a look to the future. Otolaryngologic Clinics of North America 1980;13(4):567–75.

Pullens B, Verschuur HP, van Benthem PP. Surgery for Ménière’s disease. Cochrane Database of Systematic Reviews 2013, Issue 2. Art. No.: CD005395.DOI: 10.1002/14651858.CD005395.pub3.

Saliba I, Gabra N, Alzahrani M et al (2015) Endolymphatic duct blockage: a randomized controlled trial of a novel surgical technique for Ménière’s disease treatment. Otolaryngol Head Neck Surg 152(1):122–129

Saliba I, et al, Protocole de Recherche, La decompression du sac endolympahtigue avec blocage du canal endolympahtique: une nouvelle methode pout le traitment de la maladie de Ménière, CHUM, Decembre 2015.

Salt AN, Plontke SK. Endolymphatic hydrops: pathophysiology and experimental models. Otolaryngol Clin North Am. 2010 Oct;43(5):971-83

Semaan MT, Megerian CA. Contemporary perspectives on the pathophysiology of Ménière's disease: implications for treatment. Curr Opin Otolaryngol Head Neck Surg. 2010 Oct;18(5):392-8

Shepard NT, Telian SA, Smith-Wheelock M. (1990) Habituation and balance retraining therapy. Neurol Clin. May; 8(2): 459-75.

Sennaroglu L, Sennaroglu G, Gursel B. Intratympanic dexamethasone, intratympanic gentamicin, and endolymphatic sac surgery for intractable vertigo in Ménière’s disease. Otolaryngol Head Neck Surg. 2001;125:537-543

Shi S, Guo P, Wang W. Magnetic Resonance Imaging of Ménière’s Disease After Intravenous Administration of Gadolinium. Ann Otol Rhinol Laryngol 2018;127:777–82.

Shojaku H, Watanabe Y, Fujisaka M, et al. Epidemiologic characteristics of definite Ménière's disease in Japan. A long-term survey of Toyama and Niigata prefectures. ORL J Otorhinolaryngol Relat Spec. 2005;67(5):305-309. doi:10.1159/000089413

Silverstein H, Smouha E, Jones R. Natural history vs surgery for Ménière’s disease. Otolaryngol Head Neck Surg. 1989;1:6-16.

Stahle J, Stahle C, Arenberg IK. Incidence of Ménière's disease. Arch Otolaryngol. 1978;104(2):99-102.

Steekelenburg Van JM, Weijnen Van A, De Pont LMH, Vijlbrief OD, Bommelje CC, Koopman JP, Verbist BM, Blom HM, Hammer S. Value of Endolymphatic Hydrops and Perilymph Signal Intensity in Suspected Ménière’s Disease. Am J Neuroradiol. 2020 Mar;41(3):529-534.

Takumida M, Bagger-Sjo¨back D, Wesa¨ll J. Three-dimensional ultrastructure of the endolymphatic sac. Arch Otorhinolaryngol. 1987;244:117-122.

Thomsen J. Placebo effect in surgery for Ménière's disease. A double-blind, placebo-controlled study on endolymphatic sac shunt surgery. Archives of otolaryngology 1981-5, 271-7.

Thomsen J, Bonding P, Becker B, Stage J, Tos M. The nonspecific effect of endolymphatic sac surgery in treatment of Ménière’s disease: a prospective, randomized controlled study comparing “classic” endolymphatic sac surgery with the insertion of a ventilating tube in the tympanic membrane. Acta Oto-laryngologica 1998;118(6):769–73

Van Steekelenburg JM, Van Weijnen A, De Pont LMH, Vijlbrief OD, Bommeljé CC, Koopman JP, Verbist BM, Blom HM and Hammer S. Am J of Neuroradiology February 2020, DOI: https://doi.org/10.3174/ajnr.A6410

Vereeck L, Truijen S, Wuyts F, Heyning P van de. Test-retest reliability of the Dutch version of the Dizziness Handicap Inventory. B-ENT 2006; 2: 75-80

Ware JE, Snow KK, Kosinski M, et al. SF-36® Health Survey Manual and Interpretation Guide. Boston, MA: New England Medical Center, The Health Institute, 1993.

Wladislavosky-Waserman P, Facer G.W., Mokri, B. & Kurland, L.T. (1984). Ménière’s disease: a 30-year epidemiologic and clinical study in Rochester, MN, 1951-1980. Laryngoscope, 94, 1098-1102

Xu J, Yi H, Li X, Chen W, Gao J. Effects of endolymphatic sac decompression combined with posterior tympanotomy with local steroids for intractable Meniere's disease. Acta Otolaryngol. 2020 Apr;140(4):258-261. doi: 10.1080/00016489.2019.1708458. Epub 2020 Feb 7. PMID: 32031482.

Yardley L, Kirby S.(2006) Evaluation of Booklet-Based Self-Management of Symptoms in Ménière Disease: A Randomized Controlled Trial, Psychosomatic Medicine 68:762–769.

15. APPENDICES

15.1 Appendix 1 - Investigator names and contact data

**Principal investigator**	
Name	H. Blom, MD, PhD
Function	ENT-surgeon
Department	Department of Otorhinolaryngology
Hospital	HagaZiekenhuis
City	The Hague
Telephone	+31 (0) 651257906
Email	h.blom@hagaziekenhuis.nl

**Coordinating investigator**	
Name	A.A. Schenck, MD
Function	PhD student
Department	Department of Otorhinolaryngology
Hospital	HagaZiekenhuis
City	The Hague
Telephone	+31 (0) 620280814
Email	j.schenck@hagaziekenhuis.nl

**Other main investigators**	
Name	J.M. Kruyt, MD
Function	ENT-surgeon
Department	Department of Otorhinolaryngology
Hospital	Bergman Clinics
City	The Hague
Telephone	+31(0)71-5262434
Email	k.kruyt@bergmanclinics.nl
Name	P.P.G. van Benthem, MD, PhD
Function	Professor of Otorhinolaryngology

	Chair Department of ORL-HNS
Department	Department of Otorhinolaryngology
Hospital	Leiden University Medical Centre
City	Leiden
Telephone	+31 71 526 2434
Email	p.van.benthem@lumc.nl
Name	Suzanne C. Cannegieter, MD, PhD
Function	Professor of Clinical Epidemiology
Department	Department of Clinical Epidemiology
Hospital	Leiden University Medical Centre
City	Leiden
Telephone	+31 71 526 1508
Email	s.c.cannegieter@lumc.nl
Name	W.B. van den Hout
Function	Health economist
Department	Department of Medical Decision Making
Hospital	Leiden University Medical Centre
City	Leiden
Telephone	+31 71 5264577
Email	w.b.van_den_Hout@lumc.nl
Name	S. Boehringer
Function	Statistician
Department	Department of Biostatistics
Hospital	Leiden University Medical Centre
City	Leiden
Telephone	+31 71 5269743

Email	s.boehringer@lumc.nl
Name	S. Hammer MD, PhD
Function	Neuroradiologist
Department	Department of Radiology
Hospital	HagaZiekenhuis
City	The Hague
Telephone	+31 6029561315
Email	s.hammer@hagaziekenhuis.nl
Name	S.P.M. Hombergen, MSc.
Function	Physiotherapist
Department	Physiotherapy, vestibular therapy
Hospital	HagaZiekenhuis
City	The Hague
Telephone	+31 70 2105488
Email	s.hombergen@hagaziekenhuis.nl
**Participating centres**	
Name	S.M. Winters, MD PhD
Function	ENT-surgeon
Department	Department of Otorhinolaryngology
Hospital	Duizeligheidscentrum, Gelre Ziekenhuis
City	Apeldoorn
Telephone	055 - 581 86 81
Email	s.winters@gelre.nl
Name	Y.E. Smulders, MD PhD
Function	ENT-surgeon

Department	Department of Otorhinolaryngology
Hospital	Beatrix Ziekenhuis
City	Gorinchem
Telephone	0183 644 237
Email	y.smulders@rivas.nl
Name	R. van der Berg, MD PhD
Function	ENT-surgeon
Department	Department of Otorhinolaryngology
Hospital	Maastricht UMC+
City	Maastricht
Telephone	043 387 5400
Email	raymond.vande.berg@mumc.nl
Name	T. Peters, MD PhD
Function	ENT-surgeon
Department	Department of Otorhinolaryngology
Hospital	Medisch Centrum Leeuwarden
City	Leeuwarden
Telephone	058 - 286 6200
Email	thomas.peters@mcl.nl
Name	A.F. Holm, MD PhD
Function	ENT-surgeon
Department	Department of Otorhinolaryngology
Hospital	Wilhelmina Ziekenhuis
City	Assen
Telephone	0592 34 00 53

Email	adriaan.holm@wza.nl

15.2 Appendix 2 – Extensive flowchart patient visits

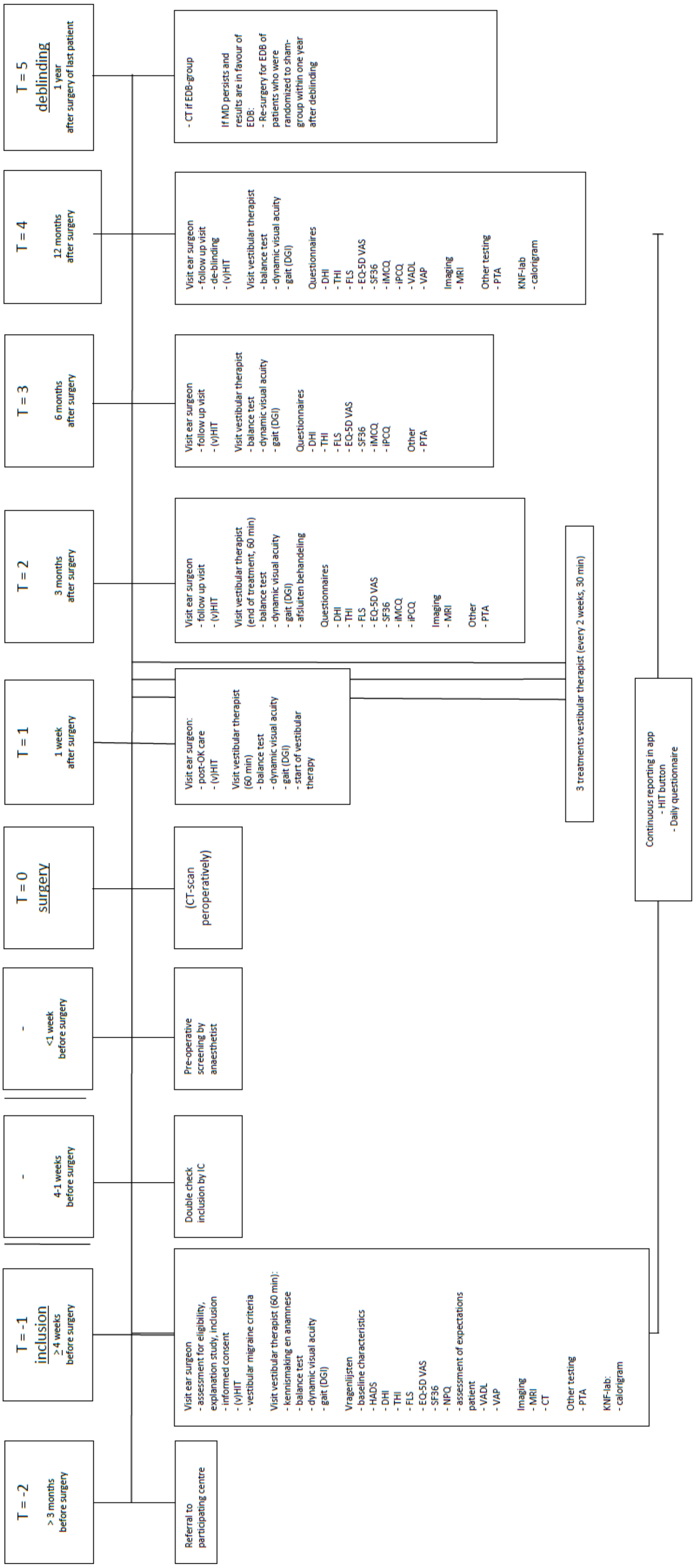

15.3 Appendix 3 – Full vestibular rehabilitation therapy

All patients in this study will receive vestibular rehabilitation after surgery. It is assumed that all patients suffer from dizziness and accompanied problems like balance, visual and gait deficits, because of inner ear hydrops and hypofunction of the labyrinth. Stable hypofunction of the labyrinth can be trained by vestibular rehabilitation (Hillier 2016) and is considered to be valuable addition to the surgery. Until now literature showed no conclusive evidence of beneficial effects of vestibular rehabilitation in patients with MD (Van Esch 2017). The lack of evidence can be attributed to the low quality of the studies, but possibly it could be less effective due to the fact that vestibular rehabilitation should be preserved for patients with stable vestibular lesions (Clendaniel 1997, Shepard 1990). In this study it can be expected the lesion is stable after surgery and vestibular rehabilitation is effective. Practical experience supports this rationale. Patient who have undergone decompression surgery could still have unstable vestibular function.

Goal of vestibular rehabilitation is to achieve a reduction of patient's specific vestibular functional deficits. The following specific goals per deficits are:

- Reduction of complaints of dizziness measured with the DHI (at least 12 points reduction) within 3 months.
- Maximize gaze stabilization (DVAT-NI), compared with norm values or reaching a plateau for 6 weeks, within 3 months.
- Optimize balance ((sharpened) Romberg, one leg stance test), in which we strive to meet norm value or reaching a plateau for 6 weeks, within 3 months.
- Optimize gait (DGI), in which we strive to have an effect of 4 or more points or reach the maximum score within 3 months.

An experienced and skilled vestibular therapist will perform a functional assessment and make an individual tailored program for each patient. Preferably the therapist is associated with one of participating centers. The therapist should be trained in vestibular rehabilitation by one of the following courses: basic course or masterclass of Apeldoorns Duizeligheidscentrum (ADC), Nederlands Paramedisch Instituut (NPI) course 'fysiotherapie bij perifeer vestibulaire duizeligheid) or NPI masterclass 'Advanced Rehabilitation for Vestibular disorders and cervicogenic dizziness. Besides training a therapist should treated at least 10 patients with vestibular rehabilitation.
All vestibular therapist will be trained in the study protocol.

- Functional assessment

A tailored vestibular rehabilitation program is given to each patient, and addresses patient functional deficits due to hypofunction of the vestibular system. This implies that the following disorders will be addressed: dizziness, visual, balance and gait disorders. There will be an intake, in which detailed history will be taken. Intensity and training form will be determined by the functional assessment. The functional assessment consists of DGI, DVAT-NI, Romberg, sharpened Romberg, One Leg Stance Test, Motion Sensitivity Quotient (see Table 1).

Table 1: Functional assessment

**Possible complaints/functional deficits**	**Assessment**
Dizziness	Motion Sensitivity Quotient
Visual disorders	Eye tracking Movement<br>Dynamic Visual Acuity – non-instrumented
Balance	Romberg (maximum 30 sec)<br>Sharped Romberg (maximum 30 sec)<br>One leg Stance test (maximum 30 sec)
Gait	Dynamic Gait Index (8 items)

- Vestibular therapy

Training exists of habituation exercises (based on the Motion Sensitivity Test, developed by Shepard & Telian, 1990), gaze stabilization exercises (according to Herdman 2014; part of Cawthorn and Cooksey), postural stabilization exercises (balance training according to the framework of Klatt (2015)) and gait training according to the framework of Klatt et al. (2015) and Clendaniel (1997). The therapy consists of the following:

- 3 times a day: habituation and gaze stabilization exercises
- 3 times a week: postural stabilization exercise and gait training
- Three 30-minute-session of vestibular rehabilitation.

For more detailed information, see table 2.

Table 2. The different component of vestibular rehabilitation

**Functional deficit**	**Modality of vestibular rehabilitation**	**Determination exercise**	**Intensity**
Dizziness<br>(induced by movements)	Habituation exercises	Movement are determined by the results of Motion Sensitivity Test; motion with	Mild to moderate dizziness (dizziness rating scale 1-2/5);<br>3-5 times, 3 times a day

		a score of 2-3 points are optional.<br>Habituation exercises are developed by Shepard & Telian (1990).<br>Each training the level of habituation will be adjusted and extended by movement with more challenging movement (amplitude/speed)	- Adjustments in program after 2 weeks<br>- Adjustment in program when patient have no complaint of dizziness during execution of the program (by telephone/mail)<br>- Moments of rest during exercise program of 10-30 sec, it's a preferable that dizziness returns to baseline level.<br>- Maximal 3 exercises<br>- After the exercise program the dizziness returns to base line level after 15-30 minuten
Visual disorders (complaints of blurry sights, visual vertigo)	Gaze stabilization exercises	- Eye tracking movement (Cawthorne & Cooksey, 1 until 3)<br><br>- Exercises of Herdman	- Addressing when this gives recognizable complaints, 3x5, 3 times a day, mild to moderate complaints<br><br>- Mild to moderate complaints, exercise for minimal 1 minute, 3 times a day.
Balance	Balance training	According to the framework Klatt (2015)	Level should be challenging; 30 sec for each part, 4-6 repetitions, daily
Gait	Gait training	According to the framework of Klatt (2015), Clendaniel 1997	4-6 repetitions, daily.
Psychogenic component (if HADS anxiety > 7)	Psychoeducation, advice	Written explanation will be given containing psychoeducation and advice of behavior change based on cognitive-behaviour theory and selfregulation theory (according to Yardley et al. 2006)	Questions of patients will be responded to in one of the consults

If a patient has an increased risk of anxiety problems (HADS > 7) a written explanation will be given containing psychoeducation and advice of behavior change based on cognitive-behaviour theory and selfregulation theory (according to Yardley 2006).

Figure 1. Schematic

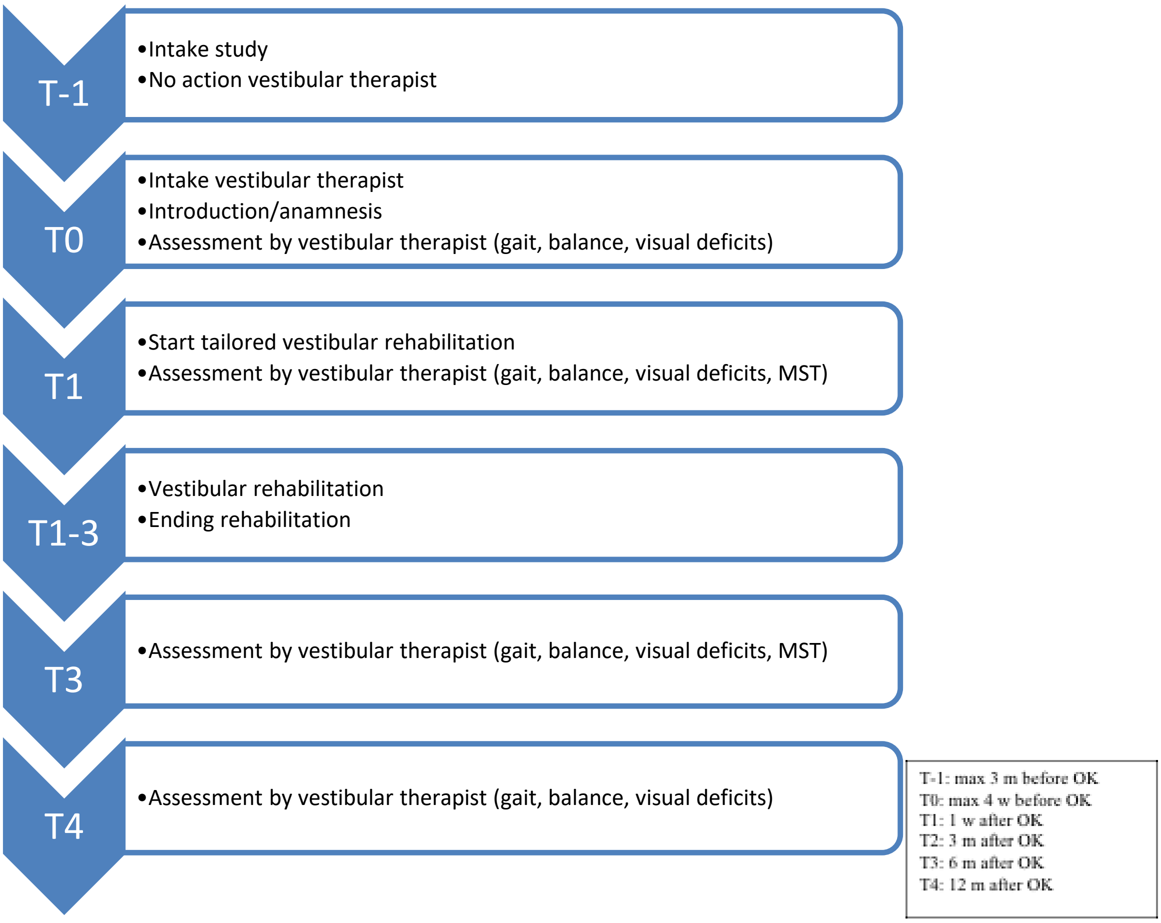

Figure 1: Functional assessment by functional deficit

1 week after surgery, the patient will visit the vestibular therapist again, to commence vestibular therapy. From that moment, the patient will start carrying out the exercises as determined by the vestibular therapist.

References

Clendaniel RA, Tucci DL.(1997) Vestibular rehabilitation strategies in Ménière's disease. Otolaryngologic clinics of North America, vol 30, nr 6, 1145-1158.

van Esch BF, van der Scheer-Horst ES, van der Zaag-Loonen HJ, Bruintjes TD, van Benthem PP. (2017) The Effect of Vestibular Rehabilitation in Patients with Ménière's Disease. Otolaryngol Head Neck Surg. Mar;156(3):426-434.

Hillier S, McDonnell M. (2016) Is vestibular rehabilitation effective in improving dizziness and function after unilateral peripheral vestibular hypofunction? An abridged version of a Cochrane Review. Eur J Phys Rehabil Med. Aug;52(4):541-56.

Klatt BN, Carender WJ, Lin CC, Alsubaie SF, Kinnaird CR, Sienko KH, Whitney SL. (2015) A Conceptual Framework for the Progression of Balance Exercises in Persons with Balance and Vestibular Disorders. Phys Med Rehabil Int; 2(4).

Shepard NT, Telian SA, Smith-Wheelock M. (1990) Habituation and balance retraining therapy. Neurol Clin. May; 8(2): 459-75.

Shepard NT, Shepard NT1, Telian SA, Smith-Wheelock M, Raj A. (1993) Vestibular and balance rehabilitation. Ann Otol Rhinol Laryngol. Mar; 102:198-205.

Shepard NT, Telian SA. (1995) Programmatic vestibular rehabilitation. Otolarnyngol Head Neck Surg. Jan; 112(1); 173-182.

Yardley L, Kirby S.(2006) Evaluation of Booklet-Based Self-Management of Symptoms in Ménière Disease: A Randomized Controlled Trial, Psychosomatic Medicine 68:762–769.

15.4 Appendix 4 – IT injection procedure

General information

Treatment with intratympanic corticosteroids is an inclusion criterion in this study. The following information describes how this treatment should be carried out.

- The patient receives at least 2 sessions of intratympanic injections.
- One session consists of at least one IT-injections
  The time between injections within one session is not determined
- One of the following substances is used
  - Dexamethasone 4-20 mg/ml
  - Triamcinolone (Kenacort) 20 mg/ml
  - Methylprednisolon (Solumedrol) 62.5 mg/nl
- Between two sessions are at least 6 weeks times

Application

Inspect the ear microscopically and assess is the tympanic membrane is intact. Apply local anesthesia. Lay the patient down and test is the tympanic membrane is anaesthetized.

With a fine needle and make a perforation in the posterior superior quadrant for venting.
Take the substance and inject +- 1 ml into the posterior inferior quadrant.
Keep the patient in horizontal position for 15 minutes, instruct them not them speak and swallow as little as possible. Explain why this is important.
Plan a consult for evaluation 6 weeks after injection.

15.5 Appendix 5 – Radiology protocol

- MR imaging

Recently developed high-resolution MRI imaging enables evaluation of the hydrops in the inner ear. Hydrops is associated with duration of MD and saccular hydrops is associated with sensorineural hearing loss (Attye 2018). Perilymphatic signal intensity is a surrogate marker for impaired blood-labyrinth permeability. Signal intensity (without) hydrops is markedly increased in the acute phase of labyrinthitis, and is increased in patients with MD (Shi, 2018). Overall, evidence is accumulating that both hydrops and signal intensity of the perilymph are associated with disease burden and may reflect disease activity.

3D Fluid Attenuation Inversion Recovery (FLAIR) sequences obtained 4 hours after 30 ml intravenous gadolinium injection delineate the perilymphatic and endolymphatic spaces of the cochlea and vestibulum separately and non-invasively. In patients with endolymphatic hydrops, the perilymphatic space surrounding the endolymph is either small or cannot be visualized. As endolymphatic hydrops is earmarked as the hallmark of Ménière's disease, the technique can used to monitor possible changes in hydrops and relate it to clinical parameters.

All patients will be imaged at a 3T MR imaging system (depending on local vendor), 4 hours after 30 ml intravenous Gadolinium (gadoterate meglumine, Dotarem. Guerbet, Aulnay-sous-Bois, France, based on previously published methods, Van Steekelenburg 2020). Patients are evaluated in the supine position with additional fixation between the patient's head and the receiver coil. Estimated duration of the MR examination is approximately 20 minutes. High-resolution T2 SPACE images of the inner ear are obtained for anatomical reference, using the following parameters: FOV 160 mm, slice thickness 0.5 mm, TR 1400 ms, TE 155 ms, number of excitations 1, flip angle 120, matrix 320 x 320, bandwith 289 Hz/pixel, turbofactor 96, voxel size: 0.5 x 0.5 x 0.5 mm and acquisition time 5 min. A 3D-FLAIR sequence will be performed using a FOV 190 mm, slice thickness 0.8 mm, TR 6000 ms, TE 177 ms, number of excitations 1, TI 2000 ms, flip angle 180, matrix 384 x 384, bandwith 213 Hz/pixel, turbofactor 28, voxelsize: 0.5 x 0.5 x 0.8 mm and acquisition time of 14 min.

If protocol requirements are insufficient in secondary centers, patients are referred to the HagaHospital for MR imaging.

All MR images are scored according to previous documented scoring methods (Bernaerts 2019, van Steekelenburg 2020), including signal intensity of the perilymph. Scoring will be

performed by two neuroradiologists (in training) twice, blinded for study occasion and definite diagnosis. If the MRI is not made in the HagaHospital, the CD containing the images will be sent through registered post to the HagaHospital, as this is the safest way of transporting patient data.

- CT imaging protocol

CT images of the temporal bone are acquired using standard clinical parameters. In short, a tube voltage of 120 kV, 230 mAs and a 0.3 mm slice thickness is used. Images are scored on visibility and anatomy of the vestibular aquaduct using Pöschl reconstructions (Juliano 2016) and are evaluated for clip location after EDB procedure.

- References

Attyé A, Eliezer M, Medici M, et al. In vivo imaging of saccular hydrops in humans reflects sensorineural hearing loss rather than Ménière's disease symptoms. Eur Radiol 2018;28:2916–22.

Baráth K et al. Detection and grading of endolymphatic hydrops in Ménière disease using MR Imaging. AJNR Am J Neuroradiol 2014;35:1387-1392.

Bernaerts A, Vanspauwen R, Blaivie C, et al. The value of four stage vestibular hydrops grading and asymmetric perilymphatic enhancement in the diagnosis of Ménière ' s disease on MRI. Neuroradiology 2019;61:421–9.

Steekelenburg Van JM, Weijnen Van A, De Pont LMH, Vijlbrief OD, Bommelje CC, Koopman JP, Verbist BM, Blom HM, Hammer S. Value of Endolymphatic Hydrops and Perilymph Signal Intensity in Suspected Ménière's Disease. Am J Neuroradiol. 2020 Mar;41(3):529-534.

Shi S, Guo P, Wang W. Magnetic Resonance Imaging of Ménière's Disease After Intravenous Administration of Gadolinium. Ann Otol Rhinol Laryngol 2018;127:777–82.

Juliano AF, Ting EY, Mingkwansook V, Hamberg LM and Curtin HD. Vestibular Aqueduct Measurements in the 45° Oblique (Pöschl) Plane. American Journal of Neuroradiology July 2016, 37 (7) 1331-133

15.6 Appendix 6 – Monitoringplan HagaZiekenhuis
